# Steviol glycosides enhance pancreatic beta-cell function and taste sensation by potentiation of TRPM5 channel activity

**DOI:** 10.1038/ncomms14733

**Published:** 2017-03-31

**Authors:** Koenraad Philippaert, Andy Pironet, Margot Mesuere, William Sones, Laura Vermeiren, Sara Kerselaers, Sílvia Pinto, Andrei Segal, Nancy Antoine, Conny Gysemans, Jos Laureys, Katleen Lemaire, Patrick Gilon, Eva Cuypers, Jan Tytgat, Chantal Mathieu, Frans Schuit, Patrik Rorsman, Karel Talavera, Thomas Voets, Rudi Vennekens

**Affiliations:** 1 Laboratory of Ion Channel Research, Department of Cellular and Molecular Medicine, KU Leuven, 3000 Leuven, Belgium; 2 TRP Research Platform Leuven (TRPLe), KU Leuven, Herestraat 49/802, 3000 Leuven, Belgium; 3 Oxford Centre for Diabetes, Radcliffe Department of Medicine, Endocrinology and Metabolism, Churchill Hospital, Old Road, Oxford OX3 7LE, UK; 4 Pôle d'endocrinologie, diabète et nutrition, Institut de Recherche Expérimentale et Clinique, Université catholique de Louvain, 1200 Brussels, Belgium; 5 Clinical and Experimental Endocrinology, Department of Clinical and Experimental Medicine, KU Leuven, 3000 Leuven, Belgium; 6 Gene Expression Unit, Department of Cellular and Molecular Medicine, KU Leuven, 3000 Leuven, Belgium; 7 Toxicology and Pharmacology, Department of Pharmaceutical and Pharmacological Sciences, KU Leuven, 3000 Leuven, Belgium

## Abstract

Steviol glycosides (SGs), such as stevioside and rebaudioside A, are natural, non-caloric sweet-tasting organic molecules, present in extracts of the scrub plant *Stevia rebaudiana*, which are widely used as sweeteners in consumer foods and beverages. TRPM5 is a Ca^2+^-activated cation channel expressed in type II taste receptor cells and pancreatic β-cells. Here we show that stevioside, rebaudioside A and their aglycon steviol potentiate the activity of TRPM5. We find that SGs potentiate perception of bitter, sweet and umami taste, and enhance glucose-induced insulin secretion in a Trpm5-dependent manner. Daily consumption of stevioside prevents development of high-fat-diet-induced diabetic hyperglycaemia in wild-type mice, but not in *Trpm5*^*−*/*−*^ mice. These results elucidate a molecular mechanism of action of SGs and identify TRPM5 as a potential target to prevent and treat type 2 diabetes.

## Abstract (web summary)

Steviol glycosides are sweet-tasting compounds isolated from a South American shrub and are increasingly used as sweeteners in foods and beverages. Philippaert *et al*. demonstrate that steviol glycosides potentiate Ca^2+^-dependent TRPM5 activity and promote glucose-induced insulin secretion and glucose tolerance.

Steviol glycosides (SGs) are found in the leaves of a scrub plant native to the subtropical regions of South America, *Stevia rebaudiana Bertoni* sp. Stevia extract has been used for centuries as a sweetening food additive in South America^1^, and was recently approved for this purpose also in the European Union and the United States. These molecules have a common aglycone core structure steviol (*ent*-13-hydroxykaur-16-en-18-oic acid), and differ in their glycosylation pattern. The two major glycosides found in the leaves are stevioside and rebaudioside A^2^. Both taste ∼200–300 times sweeter than sucrose. Non-caloric sweeteners are increasingly introduced into common foods such as diet sodas, cereals and desserts, as sugar replacement, and are for this reason recommended for weight loss and for individuals suffering from glucose intolerance and diabetes^3^. However, stevia extracts might have direct insulinotropic and anti-hyperglycaemic effects in type 2 diabetic animal models^4^. Mechanistic insight into the biological and health effects of Stevia extract and SGs is lacking.

Taste perception in type II taste receptor cells relies on the activity of transient receptor potential cation channel subfamily melastatin member 5 (TRPM5), a monovalent cation channel, activated by a rise in intracellular calcium^5^. In short, type II taste receptor cells detect sweet, bitter and umami tastants on the tongue. Taste specificity of the cell is accomplished by expression of a specific G-protein-coupled receptor. Sweet, bitter and umami taste receptors have a common downstream signal transduction cascade, which consists of the activation of phospholipase C, release of Ca^2+^ from IP_3_-sensitive Ca^2+^ stores and activation of TRPM5 (ref. 6). TRPM5 activity depolarizes the cell, which triggers the voltage-gated channel calcium homeostasis modulator 1 that mediates the release of ATP neurotransmitter, which in turn activates afferent signalling to the brain^7^. TRPM5 is also present in insulin-producing β-cells in the pancreas and in peripheral enteroendocrine cells in the gut^5^,^8^,^9^,^10^,^11^,^12^. We showed recently that TRPM5 determines the frequency of glucose-induced calcium oscillations of insulin-releasing pancreatic β-cells^10^. Consistent with the cellular expression and action of TRPM5, the systemic phenotype of *Trpm5*-deficient mice includes loss of sweet, bitter and umami taste responsiveness^13^, and decreased glucose tolerance due to impaired glucose-induced insulin release^10^,^14^. It is also interesting to note that Trpm5 expression in the small intestine from diabetic patients is negatively correlated with blood glucose concentrations^15^. Likewise, WFS-1 diabetic pancreatic islets show a reduced *Trpm5* expression^16^. Moreover, TRPM5 variants are associated with a prediabetic phenotype in humans. TRPM5 single-nucleotide polymorphisms occurred more often in patients with disturbed insulin secretion, elevated plasma glucose, lower glucagon-like peptide 1 (GLP-1) levels and decreased insulin sensitivity^17^. Specific TRPM5 polymorphisms showed increased prevalence in patients with obesity-related metabolic syndrome^18^. On the basis of these observations, the hypothesis was developed that TRPM5 might be a pharmacological target for the development of a novel type of insulin secretagogue^19^,^20^,^21^. However, since pharmacology for TRPM5 is currently limited to fairly non-selective-blocking agents, this hypothesis remained untested^22^,^23^,^24^,^25^.

Our current results indicate that SGs potentiate TRPM5 ion channel activity. In this way, they potentiate the glucose-induced insulin secretion (GIIS) from pancreatic islets and enhance peripheral taste responsiveness in type II taste receptor cells. Chronic stevioside consumption effectively prevents development of high-fat-diet (HFD)-induced diabetes in mice, in a TRPM5-dependent manner.

## Results

### Steviol and its glycosides potentiate TRPM5

**Figure 1:**
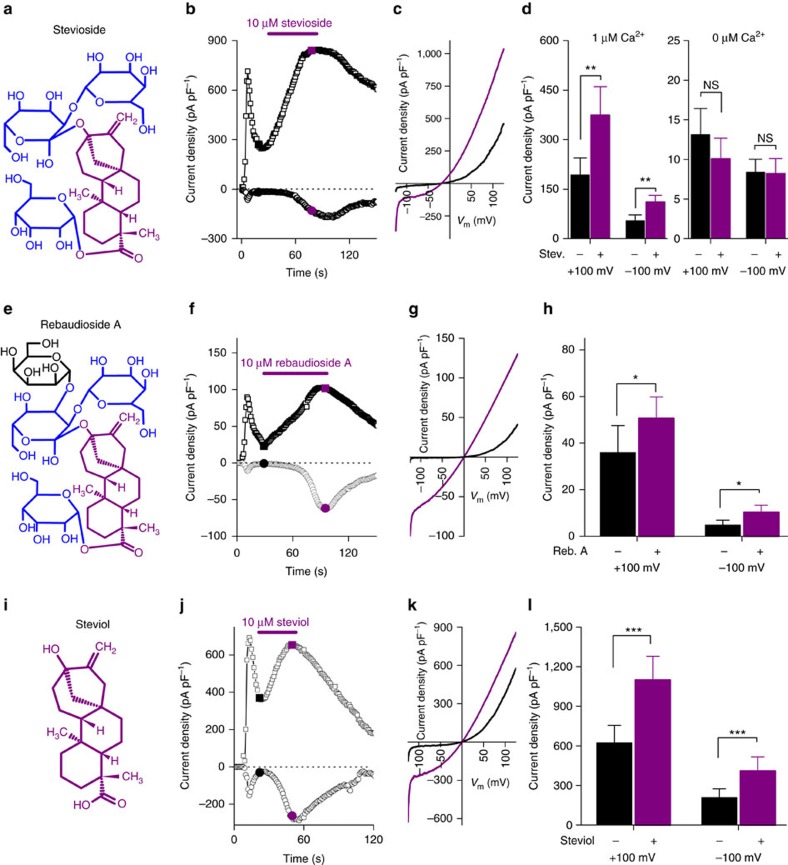
TRPM5-mediated currents are potentiated by stevioside and rebaudioside A and their aglycon steviol. (**a**) Structure of stevioside. (**b**) Time course of inward and outward currents from TRPM5 overexpressing HEK cells. To activate TRPM5, pipette solution contained 1 μM free Ca^2+^. Time 0 indicates break-in into the cell. Stevioside was applied as indicated. (**c**) Representative current traces from the time points indicated in **b**. (**d**) Left: the average±s.e.m. (*n*=13 cells, paired *t*-test, *P*<0.01) peak inward and outward current before and during the application of stevioside with 1 μM intracellular Ca^2+^. Right: the average±s.e.m. inward and outward current before and during the application of stevioside in the absence of free intracellular Ca^2+^ (*n*=10 cells, *P*>0.2, paired *t*-test). (**e**) Structure of rebaudioside A. (**f**) A time trace with inward and outward currents from *Trpm5* overexpressing HEK cells. Rebaudioside A was applied as indicated. (**g**) Current traces upon the application of rebaudioside A as indicated in **f**. (**h**) Average±s.e.m. (*n*=14 cells, paired *t*-test *P*<0.05) inward and outward current before and during application of rebaudioside A. (**i**) Structure of steviol. (**j**) A time trace with inward and outward currents from *Trpm5* overexpressing HEK cells. Steviol was applied as indicated. (**k**) Current traces upon the application of steviol as indicated in **j**. (**l**) Average±s.e.m. (*n*=12 cells, paired *t*-test *P*<0.001) inward and outward current before and during application of steviol. See also Supplementary Figs 1 and 2. Reb. A, rebaudioside A; Stev., stevioside; NS, not significant.

To test whether SGs affect TRPM5-mediated signal transduction pathways, TRPM5 currents were measured in HEK293T cells using the whole-cell patch-clamp technique. TRPM5 currents are readily activated upon break-in with 1 μM free Ca^2+^ in the pipette solution and inactivate quickly with a variable time course^25^. Instead, when cells were perfused with stevioside during the inactivating phase, a strong increase of the inward and outward current was observed, which disappeared upon washout (Fig. 1a–d). With strongly buffered [Ca^2+^]_int_, stevioside does not activate TRPM5 (Fig. 1d). Similar results were obtained with rebaudioside A, another SG that differs from stevioside by one additional glycosylation (Fig. 1e–h). We also tested steviol, the chemical core group of steviol compounds and the circulating metabolite in blood plasma after oral consumption of SGs^1^ (Fig. 1i). As shown in Fig. 1j–l, steviol yielded similar results compared to its glycosylated derivatives, indicating that the steviol group is sufficient for the interaction between SGs and TRPM5.

In cell-free inside-out patches, TRPM5 can be activated by direct application of Ca^2+^ to the intracellular side of the membrane^25^. In this setting, TRPM5 currents quickly and irreversibly inactivate^25^. Stevioside and steviol delay inactivation of Ca^2+^-activated TRPM5 currents, both when applied on the intra- and extracellular side of the membrane (Supplementary Fig. 1). These data strongly indicate that steviol interacts directly with the TRPM5 protein.

We have shown before that TRPM5 is weakly voltage-dependent^25^,^26^. As shown in Supplementary Fig. 2a,b, stevioside induces a shift of the voltage-dependent activation of TRPM5 towards more negative membrane potentials. The voltage for half maximal activation (*V*_1/2_) shifted from +145.9±33.9 to +42.8±30.6 mV in the presence of stevioside (*P*=5.1 × 10^−4^, *n*=8, paired *t*-test, Supplementary Fig. 2c). Finally, stevioside has no effect on untransfected cells (Supplementary Fig. 2d,e), and stevioside and steviol have no effect on cells expressing TRPM4 and other, more distantly related, TRP channels (TRPA1, TRPV1, TRPM3 and TRPM8; Supplementary Fig. 2f–h).

### Stevioside increases action potential frequency in β-cells

**Figure 2:**
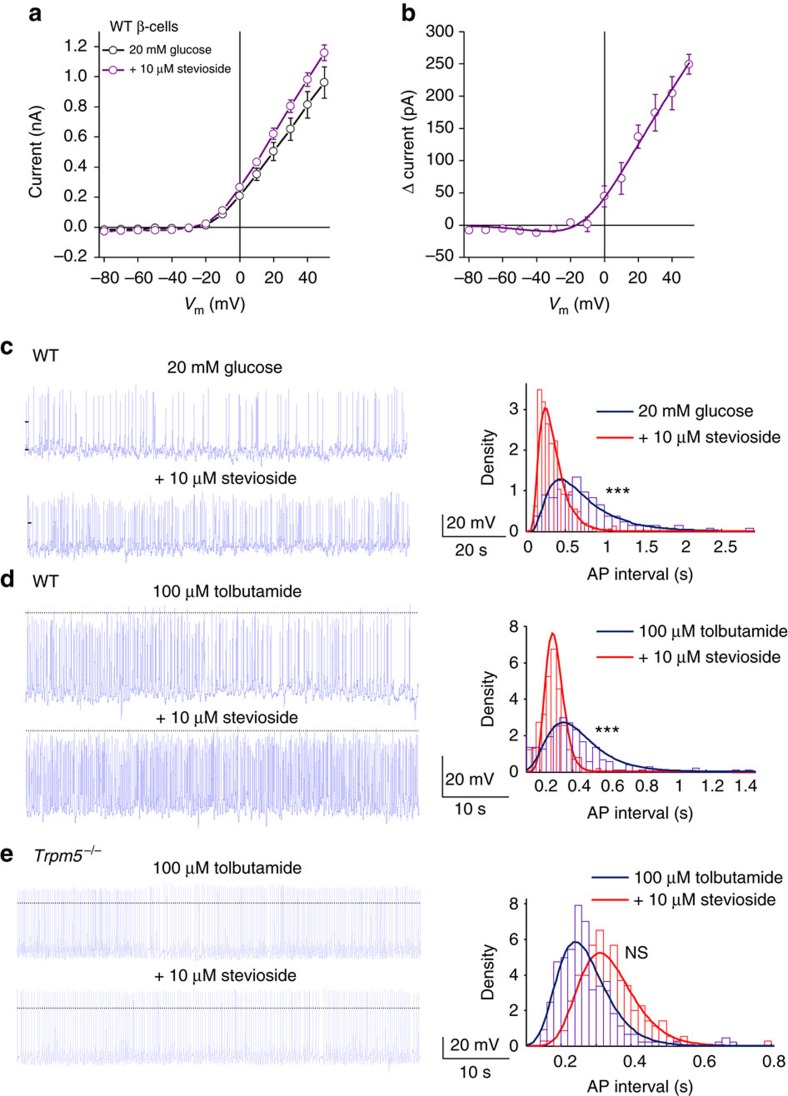
Patch-clamp recordings in isolated β-cells. (**a**) The current recorded in primary WT β-cells evoked by 20 mM glucose or 20 mM glucose+10 μM stevioside (average±s.e.m.; *n*=5 cells). (**b**) Difference current of the traces in **a**, highlighting the stevioside contributed fraction of the total current. (**c**) Left: action potentials (APs) evoked in WT β-cells by 20 mM glucose (top) or 20 mM glucose+10 μM stevioside (bottom). Right: probability density function plot depicting change in AP firing frequency properties. AP interval (s) represents the duration of the interval between APs. Density represents the proportion of intervals between APs that lie within the bin duration, normalized to account for differing bin width. (**d**) As **c** but with 100 μM tolbutamide instead of 20 mM glucose. (**e**) APs evoked in *Trpm5*^*−/−*^ β-cells in the presence of 100 μM tolbutamide or 100 μM tolbutamide+10 μM stevioside, as in **c** (*n*=4 cells). NS, not significant.

In pancreatic β-cells, TRPM5 mediates a depolarizing current, which tunes [Ca^2+^]_int_ and *V*_m_ oscillation frequency after glucose stimulation^10^. Consistent with this, a glucose-induced cation current is potentiated by stevioside in pancreatic β-cells. This current shares some properties of TRPM5, including an outwardly rectifying *I*–*V* relation and a reversal potential around 0 mV (Fig. 2a,b). We analysed the effects of stevioside on the frequency of action potential firing, stimulated by high glucose (20 mM) or tolbutamide (100 μM). Stevioside increased the frequency of action potentials in wild-type (WT) β-cells, as demonstrated by a reduction in the number of longer intervals between action potentials (Wilcoxon sign-rank test, Fig. 2c,d, *n*=5 cells each). In contrast, in *Trpm5*^*−/−*^ β-cells, the frequency of (tolbutamide-induced) action potential firing was not significantly affected by stevioside (Fig. 2e).

### Steviol and stevioside increase calcium activity in islets

**Figure 3:**
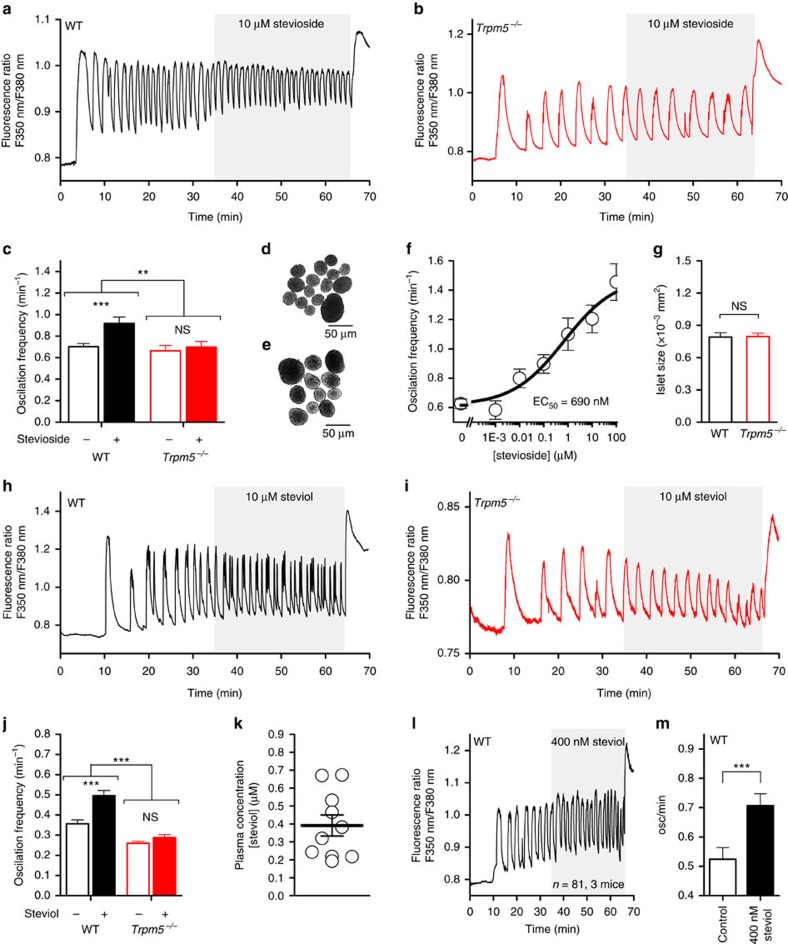
Stevioside and steviol potentiate calcium oscillations in pancreatic islets. (**a**) Representative time course of 10 mM glucose-induced Ca^2+^ oscillations in pancreatic islets isolated from WT mice, with the application of stevioside as indicated. (**b**) Same as in **a**, but from *Trpm5*^*−/−*^ islets. (**c**) Average±s.e.m. oscillation frequency of WT (*P*=1.6 × 10^−6^, *n*=71 islets from four mice, paired *t*-test) and *Trpm5*^*−/−*^ (*P*=0.15, *n*=68 islets from five mice) islets in the presence of 10 mM glucose alone or 10 mM glucose supplemented with 10 μM stevioside. Note the difference in the effect of stevioside on oscillation frequency between WT and *Trpm5*^−/−^ islets (*P*=2.63 × 10^−4^, two-sample *t*-test). (**d**) Wide-field image of WT pancreatic islets before the fluorescence measurement. (**e**) Image of *Trpm5*^*−/−*^ islets before the experiment. (**f**) Dose–response relation of different concentrations of stevioside on the potentiation of calcium oscillation frequency. The average±s.e.m. oscillations per minute during the application of 0, 1 nM to 100 μM stevioside in 10 mM glucose (269 islets from nine mice). The black line represents the logistic fit of the data, and the effector concentration for half-maximum response (EC_50_)=690 nM. (**g**) Islet size of WT and *Trpm5*^*−/−*^ islets (*n*=90 WT and 102 *Trpm5*^*−/−*^ islets). (**h**) Time course of calcium oscillations in WT islets with perfusion of steviol. (**i**) Calcium oscillations in islets from *Trpm5*^*−/−*^ mice upon perfusion with steviol. (**j**) Average±s.e.m. oscillation frequency of WT and *Trpm5*^*−/−*^ islets (WT: *P*=5.5 × 10^−15^, paired *t*-test, *n*=150 islets from four mice; *Trpm5*^*−/−*^: *P*=0.31, paired *t*-test, *n*=48 islets from two mice) in the presence of 10 mM glucose or 10 mM glucose supplemented with 10 μM steviol. (**k**) Steviol concentration in the plasma of mice exposed to 124 μM steviol in their drinking water for 3 weeks, as determined by high-performance liquid chromatography. (**l**) Example trace of a WT islet exposed to G10 and a physiological concentration of 400 nM steviol. (**m**) Calcium oscillation frequency in WT islets is significantly potentiated with 400 nM steviol (paired sample *t*-test). See also Supplementary Figs 3 and 4. NS, not significant.

When stimulated with 10 mM glucose, pancreatic islets respond with oscillations of [Ca^2+^]_int_ (Fig. 3). Application of stevioside resulted in a dose-dependent increase of the frequency of glucose-induced Ca^2+^ oscillations in WT islets, but not in *Trpm5*^*−/−*^ islets (Fig. 3a–f). Stevioside exerts this effect with an effector concentration for half-maximum response of 690 nM. Similar results were obtained with steviol (Fig. 3h–j). Note that stevioside did not induce any changes in [Ca^2+^]_int_ in low-glucose conditions (3 mM; Supplementary Fig. 3a). The frequency of glucose-induced Ca^2+^ oscillations is stable for up to 1 h in the continuous presence of glucose (Supplementary Fig. 3b,c). From these experiments it can be also derived that the size of pancreatic islets, reflecting the β-cell mass, is not statistically different between WT and *Trpm5*^−/−^ mice (Fig. 3d,e,g).

Using Fourier analysis, the effect of steviol and stevioside on WT islets can be clearly distinguished from the effect on *Trpm5*^*−/−*^ islets (Supplementary Fig. 3g–k). The Fourier-transformed (FT) frequency spectrum from WT islets highlights a shift of the frequency count towards higher frequencies, in response to stevioside unlike in *Trpm5*^*−/−*^ islets (Kolmogorov–Smirnov test, *P*=2.96 × 10^-4^, Supplementary Fig. 3g). Allocation of the islets to slow-, fast- or mixed-oscillating populations (as described in ref. 10) resulted in a significant increase of the proportion of fast-oscillating islets in the presence of stevioside (Fisher's exact test, *P*=0.009, Supplementary Fig. 3h) or steviol (Supplementary Fig. 3i). In the *Trpm5*^*−/−*^ mice, stevioside or steviol did not change the proportion of fast-oscillating islets (Fisher's exact test, *P*=0.16, Supplementary Fig. 3g,j,k). These findings demonstrate that SGs increase the frequency of glucose-induced calcium oscillations in pancreatic islets in a TRPM5-dependent manner.

When ingested by humans or mice, stevioside is metabolized in the intestine to its aglycon steviol. Once taken up in the blood, steviol circulates in the blood plasma and is finally metabolized by the liver to steviol glucuronide, which is excreted via the urine^27^. To increase the physiological relevance of our results, we measured the concentration of free steviol circulating in the plasma of mice that received 100 mg l^−1^ (124 μM) stevioside in their drinking water for 2 weeks. Circulating plasma steviol levels amounted to 391±58.6 nM in these animals (Fig. 3k)^28^. Application of this physiological concentration of steviol evoked a significant increase of glucose-induced calcium oscillation frequency, similar to stevioside (Fig. 3l,m).

It has been suggested previously that stevioside activates the sweet taste receptor, TAS1R2,TAS1R3 (refs 29, 30). Since pancreatic β-cells functionally express these receptors^31^, we tested the effect of stevioside in sweet receptor knockout mice (*Tas1r2,Tas1r3*^*(−/−)2*^). Qualitatively similar results were obtained compared to WT islets upon perfusion with stevioside or steviol (Supplementary Fig. 3d–f), that is, a significant increase (+29%) in the glucose-induced Ca^2+^ oscillation frequency (Supplementary Fig. 3f) such as WT islets (+31%, Fig. 3c). As in WT islets, the distribution of dominant oscillation frequencies of the islets from *Tas1r2,Tas1r3*^*(−/−)2*^ mice was significantly shifted towards faster frequencies in the presence of stevioside (*P*=1.07 × 10^−8^, Supplementary Fig. 3g, blue). Subdivision of the islets in slow, mixed or fast islets showed that the fast sub-population appears or increases in the presence of stevioside or steviol (Supplementary Fig. 3l,m).

TRPM5 was previously shown to play a role in GLP-1-mediated insulin secretion^32^. The observed effects of stevioside are however independent from GLP-1 receptor (GLP-1R)-mediated signalling. Stevioside has an additive potentiating effect on the GLP-1 potentiated calcium oscillation frequency (Supplementary Fig. 4a,d). As anticipated from Shigeto *et al*.^32^, the GLP-1 potentiation of glucose-induced calcium oscillations was significantly lower in *Trpm5*^*−/−*^ islets, and no additional stevioside-mediated potentiation was observed (Supplementary Fig. 4b,e). The GLP-1R antagonist exendin-3 (100 nM) inhibited the GLP-1-mediated increase in glucose-induced calcium oscillations in WT mice, but did not interfere with the stevioside-induced potentiation of calcium oscillations (Supplementary Fig. 4c,f).

### Stevioside stimulates insulin secretion *in vitro* and *in vivo*

**Figure 4:**
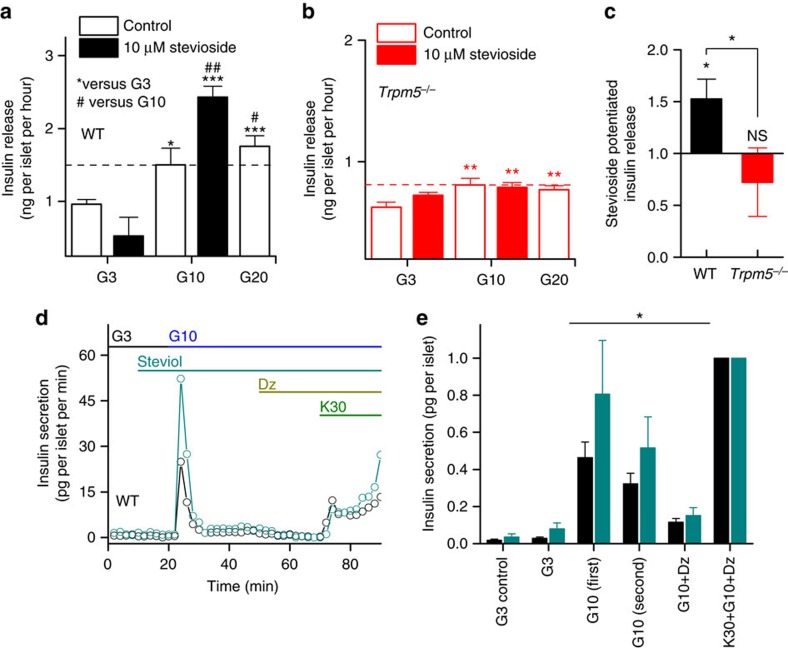
Stevioside potentiates glucose-induced insulin secretion *in vitro* and *in vivo.* (**a**) Insulin release measured *in vitro* on individual isolated islets of WT and (**b**) *Trpm5*^*−/−*^ mice (average±s.e.m. from *n*=24–30 islets per condition from four mice per genotype, two-sample *t*-test). The horizontal dashed line represents the level of the (10 mM) GIIS. (**c**) The effect of stevioside administration on glucose-induced insulin release in plasma from eight WT and nine *Trpm5*^*−/−*^ mice (average±s.e.m). (**d**) Example trace of insulin secretion during perifusion experiments without steviol (black) or with steviol supplementation at 10 min in 3 mM glucose (G3), 10 mM glucose (G10) and with 100 μM diazoxide (Dz) and 30 mM KCl (K30). (**e**) Total insulin secretion in different conditions relative to the K30 stimulus. First GIIS is the initial insulin peak between 20 and 30 min in **c** and the second phase between 30 and 50 min. Significant difference between control (*n*=6 experiments) and steviol conditions (*n*=6) of 866 islets from 22 WT mice with a two-way analysis of variance. See also Supplementary Fig. 5 and Supplementary Table 1. NS, not significant.

Considering the effect of SGs on glucose-induced Ca^2+^ oscillations, and the observation that Ca^2+^ oscillation frequency correlates with insulin release^10^, we aimed to test the effect of these compounds on GIIS. As shown in Fig. 4a,b, stevioside potentiated GIIS in WT islets, but not in *Trpm5*^*−/−*^ islets (WT: *P*=0.001, *Trpm5*^*−/−*^: *P*=0.35; two-sample *t*-test). Both in WT and *Trpm5*^*−/−*^ islets, stevioside treatment was without effect on insulin release in low-glucose conditions (3 mM). During supraphysiological saturating conditions (30 mM KCl or 20 mM glucose), the effect of steviol supplementation on insulin secretion was no longer apparent (Supplementary Table 1). Also *in vivo*, after intraperitoneal (i.p.) injection of glucose alone or glucose plus stevioside in fasted anaesthetized mice, there is a significantly higher plasma insulin level 30 min post injection of stevioside in the WT mice. This effect was equally absent in *Trpm5*^*−/−*^ mice (Fig. 4c).

In perifusion experiments, we analysed the effect of steviol on the dynamics of GIIS (Fig. 4d,e; Supplementary Fig. 5). Steviol supplementation has no effect in low glucose. The 10 mM GIIS displays a rapid peak phase (phase 1), and a more sustained level of insulin release (phase 2). Insulin secretion during both phases is increased in the continuous presence of steviol (Fig. 4d,e). Secretion was normalized to the secretion during maximal stimulation by K30, which was not different between control and steviol perfused conditions (control: 0.12±0.06 versus steviol: 0.15±0.04 pg per islet per minute, *P*=0.46; two-sample *t*-test). These effects were not observed in *Trpm5*^*−/−*^ islets (Supplementary Fig. 5a,b). Also, in moderately high-glucose conditions (15 mM), supplementation of 10 μM steviol reversibly potentiated the GIIS (Supplementary Fig. 5c,d).

### Steviol and stevioside intensify taste perception

**Figure 5:**
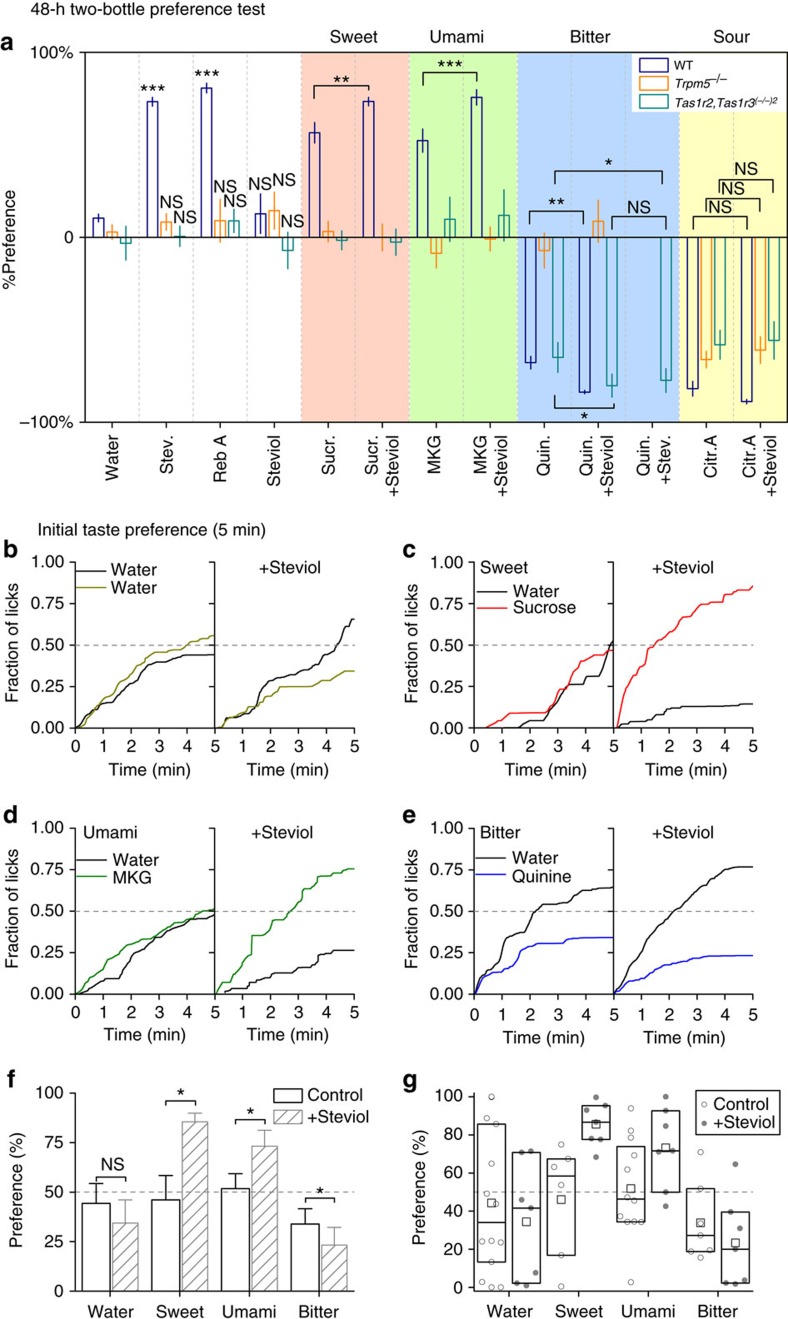
Two-bottle taste preference test. Preferences indicated are measured against water for sweet (1% sucrose), umami (150 mM monopotassium glutamate—MKG), bitter (100 μM quinine—25 μM quinine for *Tas1r2,Tas1r3*^*(−/−)2*^ in **a**) or sour (10 mM citric acid) with or without 124 μM steviol or SG supplemented to the drinking solution. (**a**) The bar graphs show either the preference (up bars) or avoidance (down bars) for the indicated compound in a two-bottle preference test during 48 h. There is clear preference for stevioside (*P*=2 × 10^−26^, *n*=48 mice over four different experiments) and rebaudioside A (*P*=2 × 10^−21^, *n*=24) in WT animals, and indifference to steviol (*P*=0.24, *n*=24, one sample *t*-test versus 0.5). Steviol increases the perception of sweet (*P*=0.037), umami (*P*=0.001) and bitter (*P*=0.003, *n*=24, paired *t*-test) solution in WT animals, but not in *Trpm5*^*−/−*^ mice. In *Tas1r2,Tas1r3*^*(−/−)2*^ mice bitter taste is potentiated by steviol (*P*=0.018) and stevioside (*P*=0.048, *n*=24, paired *t*-test). The indicated significances show a difference between the taste compound and the taste compound together with steviol. (**b**) Initial taste preference presented as normalized cumulative lick count for water versus water without (left) or with steviol (right) over 5 min after 23 h of water deprivation of 14 and 7 WT mice, respectively. (**c**) As **b** with sucrose, (**d**) MKG and (**e**) quinine. (**b**–**e**) Details from Supplementary Fig. 7a,b,e–j. (**f**) Preference from **b**–**e** represented as average±s.e.m. of relative lick count between mice for the bottle containing the tastant without or with steviol. Two-sample *t*-test (steviol/MKG) or paired sample *t*-test (sucrose/quinine). (**g**) Individual preferences from the data in **f**, indicating the variation between mice. The boxplot gives the 25–75% data interval with the mean indicated as a square and the median as a line. See also Supplementary Figs 6–9. Citr. A, citric acid; NS, not significant; Quin., quinine; Stev., stevioside; Sucr., sucrose.

To establish the *in vivo* relevance of TRPM5 modulation by SGs, we first considered taste perception. TRPM5 is an essential component of the intracellular signal transduction cascade elicited by sweet, bitter and umami tastants in type II taste receptor cells^6^. The two-bottle preference test is a well-established method to detect preferences in mice^33^, and can be combined with lick detection (lick-o-meter). We used this assay to assess the effect of steviol and SGs in WT, *Trpm5*^*−/−*^ and *Tas1r2,Tas1r3*^*(−/−)2*^ mice at various time scales. Considering that SGs are ≈ × 300 sweeter than sucrose (in humans), we used a concentration of 124 μM (equimolar to 300 times <1% sucrose) for each of the SGs and steviol. At this concentration, WT mice show a significant preference for stevioside and rebaudioside A in a 48 h two-bottle preference test (versus water; Fig. 5a, blue bars), which can be attributed to the sweet taste of the SGs. Steviol, on the other hand, does not evoke a specific preference or avoidance in WT mice (Fig. 5a,b). However, when combined with a sweet (1% sucrose), umami (150 mM monopotassium glutamate (MKG)) or bitter (100 μM quinine) tastant, steviol significantly increases the preference for sucrose and umami, and avoidance of quinine (Fig. 5a). Importantly, the increased taste preference or avoidance is apparent within 5 min, in a lick-o-meter test (Fig. 5b–g) and persistent after 60 min (Supplementary Figs 6 and 7). Steviol has no effect on the avoidance of 10 mM citric acid both in short- or long-term exposure tests. *Trpm5*^*−/−*^ mice are deficient in the signalling pathway downstream of the taste receptor. This is reflected in the lack of preference or avoidance for sweet, bitter or umami tastants (Fig. 5a, orange bars; Supplementary Figs 6 and 8). Likewise, there is no preference for SGs in *Trpm5*^*−/−*^ mice. Neither could we observe an increase in peripheral taste responsiveness upon addition of steviol to the test solution, but avoidance of citric acid was unaffected.

To test whether the taste-enhancing effect of stevioside and steviol is dependent on the sweet taste receptor, we used *Tas1r2,Tas1r3*^*(−/−)2*^ mice. It should be noted that these mice also do not detect umami tastants, as TAS1R3 is an essential subunit of the umami receptor^13^. Bitter perception is preserved in this mouse line. Interestingly, we found that both steviol and stevioside increase the avoidance of quinine in *Tas1r2,Tas1r3*^*(−/−)2*^ mice (Fig. 5a, green bars; Supplementary Figs 6 and 9). The avoidance of sour taste is conserved and not changed with steviol addition also in this mouse line.

### Oral stevioside administration decreases hyperglycaemia

**Figure 6:**
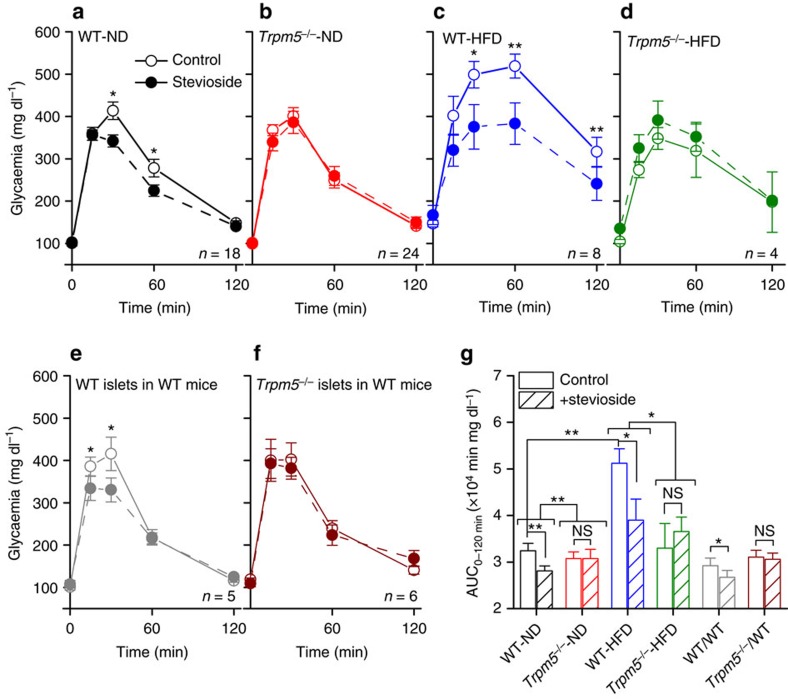
Acute treatment of stevioside lowers the glycaemia of WT mice during a GTT in a β-cell-dependent way. (**a**–**f**) Results of crossover tandem GTTs performed on the same mice with a 2 week interval. Mice received either 0.5 mg g^−1^ stevioside applied per os 2 h before the GTT, or vehicle. Individual points represent the average±s.e.m. and the significances are the result of a paired sample *t*-test of the values at the same time point; non-significant differences are not indicated. (**a**) WT mice on a normal diet, (**b**) *Trpm5^−/−^* mice on a normal diet, (**c**) WT mice 20 weeks on a HFD, (**d**) *Trpm5^−/−^* mice 20 weeks on a HFD, (**e**) WT mice with WT islets transplanted under the kidney capsule and (**f**) WT mice with *Trpm5^−/−^* islets transplanted under the kidney capsule. (**g**) Average±s.e.m. Area under the curve (AUC) from the experiments in **a**–**f**. Paired sample *t*-test within groups, two-sample *t*-test between groups. See also Supplementary Figs 10 and 11. NS, not significant.

To analyse the effects of stevioside on glucose metabolism in conscious mice, we performed two i.p. glucose tolerance tests (GTTs) in the same mice, combined with stevioside or vehicle treatment, 2 weeks apart. Blood glucose values were significantly reduced after stevioside treatment in WT, but not *Trpm5*^*−/−*^ mice (Fig. 6a,b,g). This effect was even more pronounced in obese WT mice, after 20 weeks of HFD, and equally absent in *Trpm5*^*−/−*^ animals on HFD (Fig. 6c,d,g). Notably, both in WT and *Trpm5*^*−/−*^ animals, we observed no acute effect of stevioside administration on the glucose level in the fasting state (Supplementary Fig. 10a). Even at very high dosage levels of intravenous (i.v.) administration, stevioside and steviol do not induce hypoglycaemia (Supplementary Fig. 10b,c).

To investigate whether the glucose-lowering effect of stevioside is dependent on TRPM5 expression in pancreatic islets, we transplanted pancreatic islets from *Trpm5*^*−/−*^ mice or WT mice underneath the kidney capsule of WT-recipient mice that were selectively depleted of their endogenous pancreatic β-cells by alloxan injection^34^ (Supplementary Fig. 11a). Hyperglycaemia in recipient mice was confirmed before implantation (day 0). After transplantation, normoglycaemia was re-established (Supplementary Fig. 11c,e, day 1). After recovery, acceptor mice showed normal glycaemic profiles and body mass (Fig. 6e,f, solid lines; Supplementary Fig. 11b–e). Only mice transplanted with WT islets showed a reduction in the glycaemia after stevioside treatment (Fig. 6e–g). This effect was like the effect in WT mice.

### Stevioside prevents glucose intolerance in mice on HFD

**Figure 7:**
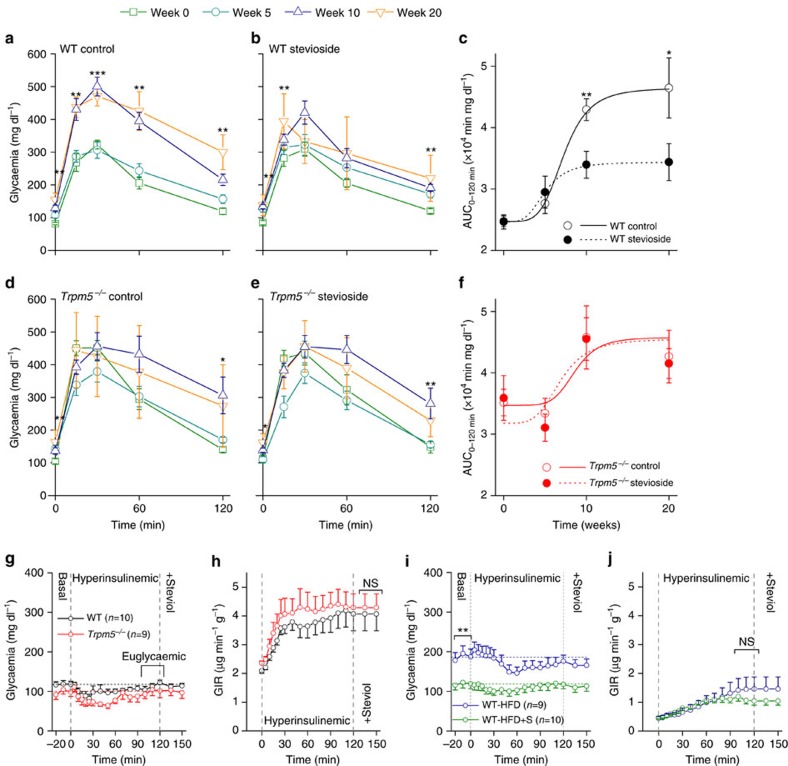
Prevention of glucose intolerance with stevioside treatment during HFD. (**a**) Results of i.p. GTT performed on eight WT mice at different time points after the start of HFD in control conditions. The indicated significance is the result of a paired sample *t*-test between week 0 and 20. Values are average±s.e.m. (**b**) Results from GTTs in eight WT mice, on HFD treated with a daily dose of 25 mg kg^−1^ stevioside. The indicated significance is the result of a paired sample *t*-test between week 0 and 20. Values are average±s.e.m. (**c**) Area under the curve (AUC) from 0 to 120 min during the GTT on the WT mice from **a**,**b**. The solid and dotted lines represent the logistic fit for the respective data sets. The represented values are averages±s.e.m. from eight animals in every group, in two independent experiments, the significances are the result of a two-sample *t*-test between the untreated and stevioside-treated groups at the same week during the HFD. (**d**–**f**) As in **a**–**c** but from *Trpm5^−/−^* mice. (**g**) Glycaemia (average±s.e.m.) during hyperinsulinemic euglycaemic clamp from WT and *Trpm5^−/−^* animals. Infusion of steviol did not change the glycaemia during euglycaemic glucose infusion. (**h**) Glucose infusion rate during hyperinsulinemic euglycaemic clamp. No significant differences were detected between WT and *Trpm5^−/−^* animals. (**i**) Glycaemia of WT mice on a HFD with or without stevioside (124 μM in drinking water) during a euglycaemic hyperinsulinemic clamp. Basal glycaemia is lower for the mice receiving stevioside, two-sample *t*-test between treatment groups on the average of −20 and −10 min glycaemic values. (**j**) Glucose infusion rates during euglycaemic hyperinsulinemic clamp experiments. Glucose infusion rate and hence insulin resistance was not different due to stevioside treatment during HFD. Acute i.v. infusion of 0.8 μmol kg^−1^ steviol did not change the glycaemia during perfusion with the euglycaemic glucose infusion rate. See also Supplementary Figs 12 and 13 and Supplementary Table 2. NS, not significant.

To test the effect of chronic stevioside treatment on the development of diet-induced diabetes, we randomly divided WT littermate mice in two groups: one group receiving HFD (control), and another group receiving HFD and stevioside. Their age, weight and glycaemic profile at the start of the experiment were not different (Fig. 7; Supplementary Fig. 12; Supplementary Table 2). Subsequent i.p. GTTs showed a time-dependent development of glucose intolerance in control mice on HFD (Fig. 7a,c). After 20 weeks on HFD blood glucose levels at 2 h after the glucose challenge, were as high as 300±53 mg dl^−1^, which is indicative for type 2 diabetes^35^. We observed a strong protective effect of stevioside against the development of HFD-induced glucose intolerance in WT mice (Fig. 7b,c). In comparison to control mice, animals after 20 weeks of HFD+stevioside had plasma levels of 220±70 mg dl^−1^ at 2 h after the glucose challenge (*P*=0.04, two-sample *t*-test compared to untreated animals). These mice maintained a normal glycaemic profile over the course of 20 weeks of HFD (Fig. 7b). In *Trpm5*^*−/−*^ animals, we observed no difference between the control and the stevioside-treated group (Fig. 7d-f). WT and *Trpm5*^*−/−*^ mice, both the control group and the stevioside-treated group, had a similar body weight and weekly caloric intake (Supplementary Fig. 12a,b,f,g). Notably, WT mice on HFD showed a significant lower resting glycaemia when treated with stevioside, which was not the case in *Trpm5*^*−/−*^ mice (Supplementary Fig. 12c,h).

The increased glucose tolerance in stevioside-treated WT animals is not due to increased islet size (Supplementary Fig. 12e). Of note, islets from stevioside-treated mice did not display the hypertrophy, which is apparent in WT HFD mice (compare Supplementary Fig. 12e (HFD and HFD+stevioside) with Fig. 3g, black (control diet)). We also tested functional responses of isolated pancreatic islets from these mice. Glucose-induced Ca^2+^ oscillations measured in islets from stevioside-treated WT mice displayed the same sensitivity to stevioside application as described above for untreated mice, while in islets from *Trpm5*^*−/−*^ mice these effects were equally absent (Supplementary Fig. 12d,i). Weight gain during 20 weeks of HFD was essentially the same between the control mice and stevioside-treated mice (Supplementary Fig. 12a,f). To study this in more detail, 20 WT mice were placed on HFD without regular GTT and associated fasting, which reduces fluctuations in the body weight. Start weight (20.7±0.2 g (control) and 20.9±0.4 g (stevioside); *P*=0.69, *n*=10 mice per group, two-sample *t*-test) and weight after 10 weeks of HFD (35.9±0.9 g (control) and 34.3±0.8 g (stevioside) *P*=0.20, two-sample *t*-test) was not different.

Insulin resistance of peripheral tissue is an important aspect of type 2 diabetes^36^. To test insulin resistance, we performed an insulin tolerance test in control WT mice, and WT mice that received 124 μM steviol for 2 weeks via the drinking water (∼0.5 mg per day per mouse). The glycaemic changes after insulin injections were not different between the two groups (Supplementary Fig. 13a). The insulin sensitivity of mice chronically fed a HFD with or without supplementation of stevioside in their drinking water was assessed in a euglycaemic hyperinsulinemic clamp test. *Trpm5*^*−/−*^ animals showed no difference in insulin sensitivity compared to WT animals (Fig. 7g,h; Supplementary Fig. 13b,c). The glucose infusion rate to reach euglycaemia in animals on a HFD was not different from the animals that were on HFD+stevioside, indicating that stevioside consumption does not alter the insulin sensitivity (Fig. 7i,j). Both groups on the HFD were, however, significantly insulin-resistant compared to normal WT animals (Fig. 7i,j versus Fig. 7g,h; Supplementary Fig. 13b,c). Note the difference in glycaemia before infusion of glucose between stevioside-treated mice and control mice (Fig. 7i). To test an acute effect of steviol on peripheral insulin sensitivity, we infused in euglycaemic conditions 0.8 nmol g^−1^ steviol i.v., while maintaining constant glucose infusion rate. This elicited no change in the glycaemia (Fig. 7g,i; Supplementary Fig. 13c,d).

To test whether stevioside influences the incretin pathway, we compared the glycaemic profile during an oral and an i.p. GTT. We observed a similar reduction of glucose levels in the stevioside-treated group during an oral GTT and an i.p. GTT (Supplementary Fig. 14a,b,e). The incretin effect, measured as the difference in area under the curve between the two GTTs, was the same when comparing the control and the stevioside-treated group (Supplementary Fig. 14d, left, *P*=0.16, *n*=10 mice per group, two-sample *t*-test). This indicates that stevioside has no impact on incretin secretion from K- and L-cells in the gut.

Finally, the gut microbiome is an important factor in the development of metabolic diseases such as diabetes^37^. We analysed this effect by treating mice with broad-spectrum antibiotics (ABX) in their drinking water, which leads to dysbiosis of the microbial colonies in the gut^38^. After 6 weeks, the effect of stevioside on the i.p. GTT was completely preserved in the ABX-treated animals. The increase of the glycaemic values after ABX treatment was the same in the stevioside-treated group as in the control group (Supplementary Fig. 14c,d,e).

### Withdrawal of stevioside leads to impaired glucose tolerance

**Figure 8:**
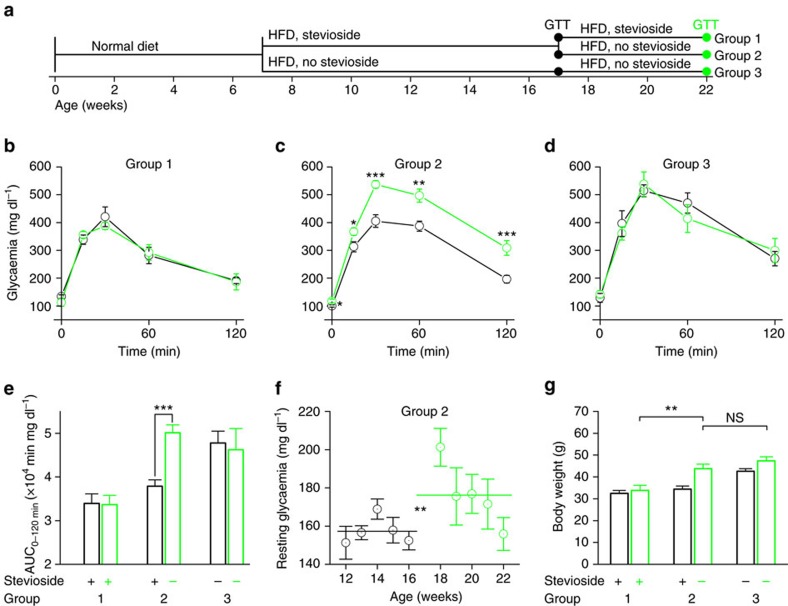
Stevioside effects are rapidly reversible during HFD. (**a**) Time line illustrating the course of the diet and stevioside treatment of three groups of mice. Time points of GTT are as indicated. (**b**) The results of the GTT after 10 weeks on HFD (black—17 weeks old) and after another 5 weeks (green—22 weeks old) of mice on HFD+stevioside (100 mg l^−1^ in drinking water). (**c**) GTTs of mice on the same time points as (**b**) with stevioside treatment terminated at the age of 17 weeks. Individual points indicate the average±s.e.m. and the significances are the result of a paired sample *t*-test at the same time point from the two GTTs. (**d**) GTTs of mice in the untreated control group at the same time points. All values are averages±s.e.m. of *n*=8–10 mice. (**e**) Average±s.e.m. area under the curve (AUC) from the experiments in **b**–**d**, paired sample *t*-test, only significant differences are indicated. (**f**) The weekly average±s.e.m. resting glycaemia (spheres) and the average over 5 weeks (lines) of the mice in group 2 during (black) and after (green) the stevioside treatment. Two-sample *t*-test between the average glycaemia during and after stevioside treatment (lines). (**g**) Average±s.e.m. Body mass of the three groups at the age of 17 weeks (black) and 22 weeks (green) before the fasting for the GTT (two-sample *t*-test). See also Supplementary Fig. 14 and Supplementary Table 3. NS, not significant.

We tested the reversibility of stevioside's effects on systemic glucose homeostasis. We assessed glucose tolerance in WT mice that received HFD in three scenarios: continuous (15 weeks) treatment with stevioside (group 1), 10 weeks of HFD+stevioside followed by 5 weeks of HFD without stevioside (group 2) and a group on HFD without stevioside for 15 weeks (control—group 3; Fig. 8a; Supplementary Table 3). Mice received 124 μM stevioside in the drinking water, as above. This experiment confirms that stevioside improves glucose tolerance in HFD-treated mice (compare group 1 and 3; Fig. 8b,d). The most striking result is discernible in group 2 where glucose tolerance deteriorates in 5 weeks after stevioside is removed from the diet to the level of the untreated group. Also, 5 weeks after stevioside treatment is stopped, resting glycaemia is significantly higher in group 2, compared to the level when stevioside was administered (Fig. 8c,e,f; Supplementary Table 3). Finally, in this experimental set-up, stevioside treatment slightly reduces HFD-induced weight gain (Fig. 8g, compare group 1, 2 and 3 at 10 weeks). Group 2 mice show an HFD-induced increase in body weight after stevioside treatment is ceased, resulting at the end of the experiment (at 15 weeks) in a similar weight as the untreated group (Fig. 8g; Supplementary Table 3).

## Discussion

Our results can be summarized as follows: (i) we show that TRPM5 is essential for the biological action of steviol and SGs; (ii) we show that potentiation of TRPM5 ion channel activity by SGs modulates taste responses and insulin release; and (iii) potentiation of TRPM5 activity protects mice against the development of HFD-induced hyperglycaemia. Our data indicate that stevioside, rebaudioside A and their aglycon steviol are novel leads for the further development of antidiabetic drugs targeting TRPM5. Considering the tremendous health cost of type 2 diabetes and that stevioside is a widely used natural product, we believe that stevioside might be a cost-effective compound for the global battle against diabetes.

Steviol, stevioside and rebaudioside A are not direct agonists of TRPM5, but potentiate Ca^2+^-dependent activity of the channel. The steviol group of SGs is sufficient for the interaction, but the interaction site on the TRPM5 protein remains unknown. Inside-out patch-clamp experiments indicate that steviol can interact with TRPM5 directly, independent of an intracellular signal transduction pathway. On the other hand, our data do not exclude other receptors for SGs, but, as outlined below, our data strongly suggest that TRPM5 is essential for the effect of SGs on taste sensation and glucose-induced insulin secretion.

**Figure 9:**
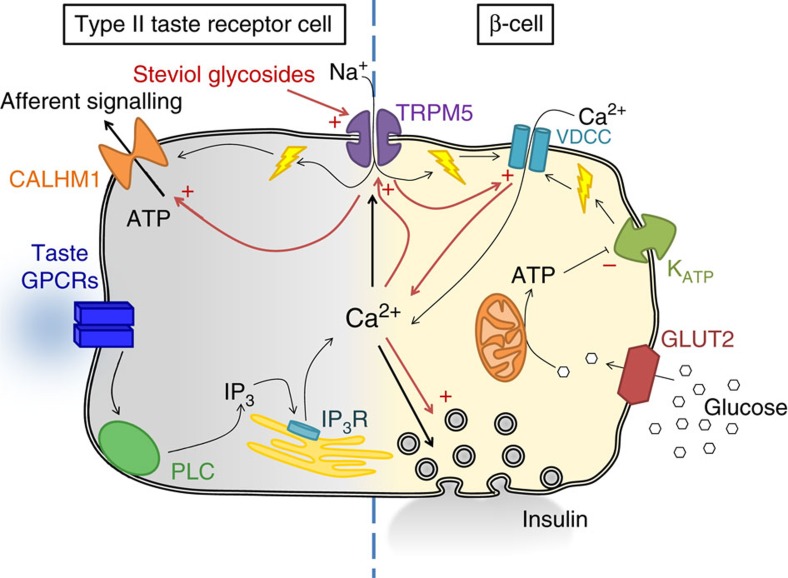
Schematic overview of intracellular pathways in type II taste receptor cells and pancreatic β-cells. Important factors in the regulation of taste perception and insulin secretion, relevant for this work, are highlighted in this figure. The effects of stevioside through TRPM5 are highlighted with red arrows. In type II taste receptor cells, positive modulation of TRPM5 increases ATP release and afferent signalling from the receptor cell. In the pancreatic β-cell, upon glucose application β-cells display parallel *V*_m_ and [Ca^2+^]_cyt_ oscillations, which drive insulin secretion. TRPM5 is active during the lag phase in between bursts of action potentials, and determines the frequency of *V*_m_ and Ca^2+^ oscillations, which modulates insulin secretion: enhanced TRPM5 activity results in a higher oscillation frequency, which results in more insulin secretion. Note that steviol and its derivatives (for example, stevioside) only enhance TRPM5 activity and insulin secretion, but that glucose is the trigger for cell signalling. Instead, sulfonylureas such as glibenclamide (not depicted) block K_ATP_ channels directly and trigger Ca^2+^ signalling and insulin secretion independent of glucose transport.

Previously, it was shown that TRPM5 is essential for sweet, bitter and umami taste detection, which are type II taste receptor cell-dependent taste modalities^6^. Stevioside and steviol specifically enhance these modalities in WT mice: avoidance of a bitter compound (quinine) and preference for sweet (sucrose) and umami (MKG). Sour taste (which is TRPM5-independent^6^) is not affected. The effect of steviol and SGs on taste is divergent, in a sense that avoidance and preference are intensified depending on the tastant. The observation that taste preference is markedly affected as short as 5 min into the preference test, excludes a significant contribution of post-ingestive effects independent of acute tasting. Taken together, our data are consistent with the model that TRPM5-mediated depolarization of type II taste receptor cells serves as the link between taste receptor activation and initiation of afferent signalling via ATP release from these cells, and that SG-mediated potentiation of TRPM5 activity potentiates this signalling pathway (Fig. 9). Our data identify steviol as a novel, potent and specific modulator of bitter, sweet and umami taste, which is taste neutral on itself. It is tempting to speculate that the extreme sweetness of SGs originates likely from a dual interaction with the sweet taste receptor but also via potentiation of TRPM5 channel activity, via its steviol core.

Steviol and SGs augment glucose-induced Ca^2+^ oscillations and insulin release in WT pancreatic islets, but not *Trpm5*^−/−^ islets. These data are consistent with the idea that TRPM5 serves as a facilitator of fast glucose-induced Ca^2+^ oscillations, which trigger insulin release (Fig. 9). This model was originally proposed from our analysis of *Trpm5*^−/−^ mice^10^, and SGs are the first compounds that can validate this hypothesis. It is worth noting that steviol and SGs yield a very similar effect as GLP-1 on glucose-induced Ca^2+^ oscillations in islets. Interestingly, Shigeto *et al*.^32^ could show that TRPM5 is directly involved in the signal transduction in the pancreatic β-cell elicited by GLP-1. This further supports the similar action of TRPM5 activators and GLP-1R agonists. Our data exclude that the GLP-1R is involved in the functional effect of steviol and SGs on pancreatic islets, as the effect of stevioside and GLP-1 is additive, and the stevioside effect is not affected by a GLP-1R blocker.

The dosage of stevioside used in the drinking water of mice in both our drinking test and during the HFD experiments was 124 μM or 100 mg l^−1^. This concentration is half of the maximum allowed dosage of SGs in soft drinks allowed in the European Union, and <25% of the suggested maximal dose by the World Health Organization and Food and Agriculture Organization^39^,^40^. Consumption of this dosage of stevioside in the drinking water resulted in a plasma concentration of 396 nM steviol in mice. We show that also at these physiological concentrations steviol has clear effect on glucose-induced Ca^2+^ signalling in pancreatic islets, indicating that the dosages of stevioside that we use are realistic and physiologically relevant.

It has been suggested that SGs interact with the mammalian sweet taste receptor, TAS1R2/TAS1R3 (refs 29, 30) and ATP-sensitive K^+^ channels in pancreatic β-cells^41^, which might explain some of the data we observe. However, our results exclude that the potentiating effect of steviol and stevioside on pancreatic islet function is mediated via the TAS1R2/TAS1R3 receptor. We observed essentially the same effect of steviol and stevioside on Ca^2+^ signalling in *Tas1r2,Tas1r3*^*(−/−)2*^, as in WT islets. Our observation that stevioside does not induce Ca^2+^ signals and insulin release in pancreatic islets in low-glucose conditions (3 mM) argues against the idea that stevioside would be a direct blocker of ATP-sensitive K^+^ channels.

Acute oral administration of stevioside reduces hyperglycaemia in a GTT in WT, but not in *Trpm5*^*−/−*^ mice. Importantly, the same effect is present in alloxan-diabetic WT-recipient mice that received WT islets, but not in WT-recipient mice that received *Trpm5*^*−/−*^ islets. This shows that the glucose-lowering effect of stevioside is dependent on the expression of TRPM5 in pancreatic islets, and not from a TRPM5-dependent action in peripheral tissue outside of the pancreatic islet.

Long-term oral intake of stevioside improves the development of plasma hyperglycaemia in WT mice on HFD, but not in *Trpm5*^*−/−*^ mice. There was no difference in the development of insulin resistance in the control and stevioside-treated group. HFD-induced obesity in mice is commonly used for the development of insulin resistance and IGT. Because obesity is induced via food-intake/composition and not by genetic manipulation, this model is considered to be relevant for the human condition. Interestingly, two studies suggest that *Trpm5*^*−/−*^ mice are resistant to diet-induced obesity; in Larsson *et al*.^42^ on a HFD and in Glendinning *et al*.^43^ on a carbohydrate-based diet. However, in both studies, *Trpm5*^*−/−*^ mice had a significantly lower caloric intake, which could explain the apparent resistance to obesity. In our study, WT and *Trpm5*^*−/−*^ mice had the same caloric intake and gained the same amount of weight on HFD.

Similar as GLP-1, stevioside treatment is weight neutral or even slightly limits weight gain during HFD. Furthermore, stevioside and steviol do not induce hypoglycaemia. Stevioside enhances the physiological profile of insulin secretion, instead of inducing increased plasma insulin levels independent of plasma-glucose concentrations, as sulfonylureas and exogenous insulin. In this respect, we propose that TRPM5-potentiating compounds might be functionally equivalent to GLP-1 mimetics. GLP-1R agonists are highly successful in diabetes care. The drawback of current GLP-1R agonists is the way of administration, as they are often injectables, the high price and the short time of action^44^. Although advances are being made in improving GLP-1R agonists, the identification of TRPM5 as a new pharmacological target with an analogous mode of action broadens the possibilities for drug development.

Considering the epidemiological development of type 2 diabetes and the financial burden on public healthcare systems worldwide, there is an eminent need to develop cost-effective pharmacological interventions for this condition. An unmet need on the anti-diabetes drug market is compounds that prevent or delay the progression of the disease. Our data show that stevioside has potent therapeutic effects for the prevention and treatment of type 2 diabetes in mice, and we show that the molecular mechanism of these effects is the potentiation of TRPM5 activity in β-cells. Thus, we anticipate that TRPM5-potentiating drugs are a bona fide target for the development of antidiabetic drugs, and that steviol and its glycosylated derivatives are interesting start compounds to develop this novel type of drugs.

## Methods

### Cell culture and transfection

HEK293T cells were obtained from American Type Culture Collection and split twice weekly. Cells were grown in Dulbecco's modified Eagle's medium containing 10% fetal calf serum, 2 U ml^−l^ penicillin and 2 mg ml^−1^ streptomycin, and 2 mM L-glutamine. Cells were transfected with *Trpm3*, *Trpm8*, *Trpa1* or *Trpv1* using Mirius transIT-293 transfection reagent.

### Animals

All animal experiments were approved by the ethical committee for animal welfare of the KU Leuven. Sample size was determined using a power analysis based on the results of preliminary experiments. Mouse strains were homozygously bred in a conventional animal facility. We used C57Bl6/J (WT) and B6;129-*Trpm5*^*tm1Csz*^/J (*Trpm5*^−/−^, obtained from C. Zuker, NY, USA) animals. The *Trpm5*^−^ allele was backcrossed for 12 generations in C57Bl6/J background. Sweet receptor knockout (*Tas1r2,Tas1r3*^*(−/−)2*^) mice were derived by crossing B6;129-*Tas1r2*^*tm1Csz*^/J and B6;129*-Tas1r3*^*tm1Csz*^/J mice (obtained from Jax Labs).

Islets for Ca^2+^ imaging were isolated from male and female mice aged 10–18 weeks, or after the diet study. Action potential recordings, static insulin secretion, insulin secretion under dynamic perfusion and *in vivo* insulin secretion (Fig. 4; Supplementary Fig. 5) was assessed in age-matched male mice. For the insulin secretion experiments in supraphysiological conditions (Supplementary Table 1), we used age-matched female mice. Drinking and licking experiments were performed on male mice aged 10–20 weeks. Donor pups for the transplantation experiments were used before visual sexing is possible and were whole-litter mixed gender. Receptor animals were 6-week-old littermate WT mice. Diet experiments were started with 7-week-old male mice. Male, 8-12 week old, WT or *Trpm5*^*−/−*^ mice or mice after the diet test were used for the euglycaemic clamp or insulin tolerance test.

### Electrophysiology

HEK293T cells stably transfected with *Trpm5* or *Trpm4* were developed using the Flp-In system (Life Technologies). The cells were seeded onto poly-L-lysine-coated coverslips and incubated for 2 h. The coverslips were mounted onto an inverted microscope equipped with a multichannel perfusion system. We used the whole-cell patch-clamp configuration. A ramp protocol from −125 to +125 mV in 300 ms, from *V*_hold_=0 mV, was applied to the cells at 1 Hz. The current at +100 and −100 mV was extracted to analyse time-dependent current changes. We measured steady-state control conditions and peak currents during the application of stevioside, rebaudioside A or steviol. Voltage dependency of TRPM5 was analysed with a voltage-step protocol, from *V*_hold_=+25 mV, consisting of a 75 ms test-pulse from −175 to +150 mV in 25 mV increments. The membrane potential was kept at −125 mV for 50 ms before returning to holding potential. The analysed current is the steady-state current at the end of the test-pulse. This was fitted with the Boltzmann equation and *V*_1/2_ was calculated in the presence of stevioside and in control conditions, of the same cells. The standard external solution contained (mM) 150 NaCl, 6 KCl, 1.5 CaCl_2_, 1 MgCl_2_, 10 glucose and 10 HEPES titrated with 1 M NaOH to a pH of 7.4. The appropriate amount of stevioside was added to this solution. Our internal solution contained (mM) 50 NaCl, 100 *N*-methyl-D-glucamine, 10 HEPES, 4 K_2_ATP, 10 ethylene glycol tetra-acetic acid and 8.62 CaCl_2_ (resulting in 1 μM of free Ca^2+^), titrated to pH 7.2 with 1 M HCl. The internal solution for measuring in the absence of intracellular calcium contained (mM) 50 NaCl, 100 *N*-methyl-D-glucamine, 10 HEPES, 4 K_2_ATP and 10 1,2-Bis(2-aminophenoxy)-ethane-*N*,*N*,*N*′,*N*′-tetra-acetic acid, titrated to pH 7.2 with 1 M HCl.

For inside-out patch recordings, the bath and pipette solution contains in (mM) 156 NaCl, 1.5 CaCl_2_, 1 MgCl_2_ and 10 HEPES at pH 7.4, supplemented with 10 μM steviol or 10 μM stevioside in the pipette as indicated. After excision, the patch is kept in a calcium-free bath solution containing (mM) 150 NaCl, 1 MgCl_2_, 10 HEPES and 10 ethylene glycol tetra-acetic acid at pH 7.3. The calcium-containing solution was (mM) 150 NaCl, 1 MgCl_2_, 10 HEPES and 0.5 CaCl_2_ at pH 7.3 supplemented with 10 μM steviol in the indicated condition. The stimulation protocol consists of a 500 ms step at −100 mV immediately followed by a 250 ms step to +100 mV at 1 Hz. The analysed currents are the steady-state currents extracted at the end of the voltage step.

Membrane potential recordings in β-cells were performed using the perforated-patch whole-cell configuration. The pipette solution contained (mM) 120 K-gluconate, 10 KCl, 10 NaCl, 1 MgCl_2_, 5 HEPES (pH 7.2 with KOH) and 0.24 mg ml^−l^ amphotericin B, while the bath solution contained (mM) 140 NaCl, 3.6 KCl, 0.5 MgSO_4_, 1.5 CaCl_2_ and 10 HEPES (pH 7.2 with NaOH). Action potential firing in isolated β-cells was induced by 100 μM tolbutamide or 20 mM glucose and recorded in the presence, or the absence, of 10 μM stevioside. The interval between subsequent action potentials was calculated and distribution was compared using a non-parametric Wilcoxon sign-rank test in Matlab (R2012a). To examine voltage dependency of the glucose-induced current, cells were held at −70 mV and stepped for 200 ms to voltages ranging from −80 to +50 mV.

### Calcium imaging on HEK cells

Cells were incubated with 1 μM FURA2-AM for 1 h and mounted on a fluorescence microscope equipped with a multichannel perfusion system. Transfected cells were identified by green fluorescent protein expression. FURA2 fluorescence was followed at 1 Hz sampling frequency. Extracellular solutions as indicated above were used. We applied 10 μM stevioside, 10 μM steviol and used specific activators of the channels of interest as positive control, that is, 2 μM capsaicin for TRPV1, 100 μM allyl isothiocyanate for TRPA1, 500 μM (+)menthol for TRPM8 and 20 μM pregnenolone sulphate for TRPM3.

### Isolation of pancreatic islets and single cells

The Krebs solution for preparation contained (in mM) 119 NaCl, 4.75 KCl, 1.2 MgSO_4_, 1.18 KH_2_PO_4_, 2.54 CaCl_2_, 5 NaHCO_3_, 20 HEPES and 5 glucose (pH 7.4). Collagenase P (2 mg ml^−l^) was injected into the pancreatic duct of C57Bl6/J WT, *Trpm5*^*−/−*^ or *Tas1r2,Tas1r3*^*(−/−)2*^ mice, followed by dissection of the pancreas. Digestion of the whole pancreas was obtained by shaking for 8 min at 37 °C. After gravitational sedimentation, islets were isolated by three rounds of handpicking. Islets were cultured in advanced RPMI 1640 (Gibco) supplemented with 10% FCS, 100 U ml^−1^ penicillin/streptomycin and 4 mM glutamax at 37 °C and 5% CO_2_. Islets were dispersed into single cells by trypsin digestion. The cells were cultured at 37 °C and 5% CO_2_ in RPMI 1640 medium supplemented with 10% FCS, 10 U ml^−1^ penicillin/streptomycin, and 10 mM glucose, for 24 h prior to experimentation.

### Calcium imaging on islets

Pancreatic islets were incubated with 1 μM FURA2-AM for 1 h in a humidified incubator at 37 °C and 5% CO_2_. They were mounted on an Olympus BX51W1 inverted fluorescence microscope equipped with an UMPLANFL × 10 objective with a Bioptech objective heater set at 37 °C. We used a Warner Instruments CL-100 temperature controlled multichannel perfusion system, kept at 37 °C. Excitation was done with a Polychrome V lichtsource and image acquisition with an Andor iXon 888 camera, pre-amplifier gain was set to 1, EM gain to 160 with the TILL Photonics Live Acquisition 2.3.0.18 software. We measured the fluorescence of whole pancreatic islets. The islets were perfused with a solution containing (mM) 120 NaCl, 4.8 KCl, 1.2 MgCl_2_, 2.5 CaCl_2_ and 10 HEPES (pH 7.4) supplemented with the indicated amount of glucose and stevioside or steviol. Depolarization of the islets with 30 mM KCl was used as positive control at the end of the measurement. Ca^2+^ oscillation frequency before or during the application of stevioside was analysed. The FT frequency spectrum of the first time derivative of the calcium oscillations was used in further analysis. The relevant oscillation frequencies between 0 and 0.05 Hz were determined. The highest peak represents the dominant oscillation frequency and all frequencies peaking at ≥60% of the maximum were considered relevant frequencies. The dominant oscillation frequency distribution during perfusion with 10 mM glucose was compared with the dominant oscillation frequency during perfusion with 10 mM glucose and 10 μM stevioside or steviol with the Kolmogorov–Smirnov test. On the basis of the oscillation frequency, the islets were divided into fast- (relevant frequencies only >0.015 Hz), slow- (relevant frequencies only <0.015 Hz) or mixed- (relevant frequencies in both parts of the FT spectrum) oscillating islets. The proportion of fast-oscillating islets in control and stevioside conditions was compared using Fisher's exact test.

### Insulin release

After isolation and overnight incubation, pancreatic islets from six mice were pooled and transferred from the culture medium to 3 mM glucose containing Krebs solution for 30 min. Individual islets were transferred to a well in a 96-well plate with the appropriate glucose and stevioside concentration^45^. After 60 min of incubation at 37 °C, the supernatant was collected. The experiment was repeated twice, and insulin concentration was determined on duplicate samples (Fig. 4a,b).

Insulin secretory capacity of the islets in G5, K30 and G20 was determined by comparing the released insulin from pooled islets to the total insulin content of these islets (Supplementary Table 1). The represented data are a result of five independent experiments (mice) per genotype.

For *in vivo* insulin release experiment, fasted WT or *Trpm5*^*−/−*^ mice were anaesthetised through an i.p. injection with 5 μl g^–1^ VDF (ventranquil:dormicum:fentanyl:water 1:2:10:3). The mice received 2.5 g kg^–1^ glucose (30% solution in H_2_O) or 2.5 g kg^–1^ glucose with 200 mg kg^–1^ stevioside at time point 0 min. Blood samples were taken at 0 and 30 min. Blood was sampled by filling heparinized capillaries through tail bleeding. The blood was centrifuged for 12 min at 5,000 r.p.m. and 4 °C, and the plasma was collected for further analysis.

In all cases, the insulin concentration was determined using the Crystal Chem Inc, USA, ultra-sensitive mouse insulin ELISA kit, as per the manufacturer's protocol.

### Dynamic insulin secretion experiments

After overnight culture in RPMI 1640 containing 10% heat-inactivated fetal calf serum, batches of 40–200 islets were perifused at 37 °C at a flow rate of 1 ml min^−1^. The perifusion medium (pH 7.4) used for these experiments contained (in mM): 124 NaCl, 4.8 KCl, 2.5 CaCl_2_, 1.2 MgCl_2_, 20 NaHCO_3_, 1 mg ml^−l^ bovine serum albumin and various test agents as indicated. It was gassed with O_2_:CO_2_ (95:5%). Effluent was collected every 2 min and insulin was measured by radioimmunoassay.

### Stevioside treatment and HFD

Only male mice were used in the diet experiments. After weaning, the mice were randomly allocated to a test group per genotype and housed individual. At the age of 7 weeks, the diet was switched from normal chow (ssniff spezialdiäten, V1535-0) to pellets containing 30% (*w/w)* saturated fat (ssniff spezialdiäten, E15116-34), given *ad libitum.* There was a weekly measuring of the body mass and the blood glucose. This resting glycaemia was taken during the light cycle of the mice with a standard glucometer by a drop of blood after tail puncturing in conscious unrestrained mice. The consumed food was measured by weighting the on the grid pellets every week. There was free access to drinking water and the supplementary drinking bottle from the indicated groups was filled daily with a stevioside containing solution (25 mg stevioside per kg in a 0.1% solution in water). The mice were monitored to consequently drink the stevioside solution. In the experiments decribed in Fig. 8 and Supplementary Fig. 14, five mice were housed in one cage. Stevioside (100 mg l^−1^) and antibiotics (ABX—1 g l^−1^ metronidazole and 200 mg l^−1^ ciprofloxacin) were added to the drinking water of the indicated groups. No animals were excluded from the analysis, outliers in resting glycaemia or food consumption at a distinct time point were attributed to experimental error and discarded from final analysis.

### Glucose tolerance test

The mice were fasted overnight in their home cage before the GTT with free access to water. Mice from different treatment groups were evenly distributed over technical replicates of the GTT. The investigator was blinded towards the treatment group the animal was allocated to. For the acute stevioside administration, the mice received either 500 mg kg^−1^ stevioside or the same volume of vehicle via oral gavage 2 h before the start of the GTT. At time 0 min, they receive an i.p. injection of 2.5 g kg^−1^ glucose in a 30% solution in water. In the case of the oral GTT, the glucose is administered through oral gavage. Glycaemia is followed at time points 0, 15, 30, 60 and 120 min using a standard glucometer (Aviva Accu-Check). The tip of the tail was punctured with a 26 G needle to initiate bleeding. After the GTT, the mice received *ad libitum* feeding of their appropriate diet. At individual time points, we show average±s.e.m. and the indicated significances are the result of a paired or a two-sample *t*-test. We excluded animals were the maximal glycaemia did not exceed 250 mg dl^−1^ from analysis. The results of the GTT are also presented as area under the curve of the glycaemia during the 120 min of the GTT.

### Euglycaemic hyperinsulinemic clamp

Mice were fasted overnight before the experiment. To obtain anaesthesia, mice under 40 g were i.p. injected with a mixture of 3.125 mg kg^−1^ acepromazine, 3.125 mg kg^−1^ midazolam and 0.156 mg kg^−1^ fentanyl while mice over 40 g received half this dose. A maintenance bolus of 18.75 μg acepromazine, 18.75 μg midazolam and 0.936 μg fentanyl was subcutaneous injected every 45 min. An i.v. catheter was placed in the tail vein and initially PBS was infused at 50 μl h^−1^ for 40 min using syringe pumps and the basal glycaemia was determined as the average measured at −20 and −10 min, before the start of the hyperinsulinemic clamp. The mice received a bolus of 2.7 mU insulin at 0 min and continuous infusion of 6.8 mU insulin per hour. In all, 12.5% glucose in PBS was i.v. infused at a variable rate to maintain euglycaemia, determined every 5–10 min by tail bleeding. After 2 h, we infused 0.8 μmol kg^−1^ steviol while maintaining the insulin and glucose infusions at the euglycaemic level.

### Islet transplantations

Islets from 2–3-week-old WT or *Trpm5*^*−/−*^ donor mice were isolated by collagenase digestion, washed and counted. Thereafter, islets were transferred to silicon microtubing (Becton Dickinson, Erembodegem, Belgium), and centrifuged for 5 min at 1,500 r.p.m. to create a compact pellet of islets for injection^46^. Endogenous β-cells from 7-week-old WT-recipient mice were selectively destroyed with 90 mg kg^−1^ alloxan 2 days before transplantation. Severe hyperglycaemia and glucosuria were confirmed in the recipient mice shortly before transplantation. During transplantation, the mice were anaesthetized with ketamine/xylazine i.p. (80–100 mg kg^−1^ ketamine and 5–10 mg kg^−1^ xylazine) and the left kidney was exposed by a lumbar incision. Recipient mice were given 500 islets under the kidney capsule. Non-fasting blood glucose levels from the tail vein of each recipient were measured to monitor functionality of the transplanted islets, 1 day, 1 week and 4 months after transplantation. Mice recovered for 14 days after surgery before a GTT was performed.

### Plasma steviol concentration determination

Ten WT mice were given 124 μM steviol in their drinking water during 21 days. Blood was collected after anaesthesia by exsanguination through puncture of the maxillary vein. The plasma was separated by 10 min centrifugation at 10,000 r.p.m. A liquid–liquid extraction was employed as follows: to 50 μl of plasma, 1 μl (1 mg ml^−1^ in MeOH) ibuprofen was added as an internal standard. The mixture was vortexed and protein precipitation was carried out adding 50 μl acetonitrile while vortexing. After centrifugation, the upper layer was collected and 25 μl was injected. Liquid chromatography–mass spectrometryanalysis was carried out using a UFLC Shimadzu system consisting of a LC-20ADXR pump, a SIL-20ACXR autosampler, a DGU-20A3 degasser and a CTO-20A oven (Shimadzu Prominence, Antwerpen, Belgium) in combination with a 3200 QTRAP (ABSciex, Halle, Belgium) and Analyst software (version 1.5). The instrument was operated in atmospheric pressure chemical ionization, negative-single ion mode monitor *m/z* 317.1 (steviol) and *m/z* 205.2 (ibuprofen). The corona discharge needle voltage was −4,500 V and heated nebulizer temperature was set at 500 °C for 0.5 ml min^−1^ flow rate. Nitrogen was set as nebulizer gas, auxiliary gas at 50 p.s.i. and curtain gas at 35 p.s.i. Other parameters were as follows: declustering potential −50 V, entrance potential −10 V. Liquid chromatography conditions: an Accucore C18 column (2.6 μm particle size, 2.1 mm × 100 mm) was used. Mobile phases were (A) 20 mM amoniumacetate at pH 4.8 and (B) acetonitrile. The used flow rate was 0.5 ml min^−1^. The autosampler temperature was set at 10 °C, the column oven at 30 °C. The method had following gradient conditions 0–0.01 min: 20% B; 0.01–10 min: 70% B; 10–11: 20% B. Retention times were defined as 7.07 (steviol) and 6.21 (ibuprofen). Calibration curves were constructed using linear regression of the peak area ratios of steviol against internal standard (ibuprofen). Calibration standards were prepared by spiking blank plasma at concentrations 10, 50, 100, 250, 500, 1,000, 2,500, 5,000 ng ml^−1^ (*R*^2^=0.9926).

### Drinking test

WT, *Trpm5*^*−/−*^ or *Tas1r2,Tas1r3*^*(−/−)2*^ mice were age-matched, individually caged and given two drinking bottles. After an adaptation period the animals drank 50% from each of the bottles containing deionized water. A test solution was placed in one of the bottles, and after 24 h, the position of the bottles was switched to control for a position preference. The bottles were weighted to measure the intake from each bottle. All tastants were given in an increasing stimulating order. The used solutions were 1% sucrose (sweet), the minimal concentration for which a preference can be detected^47^. A stimulatory concentration of 150 mM MKG; umami)^48^, 100 μM quinine (bitter) or 25 μM in the *Tas1r2,Tas1r3*^*(−/−)2*^ mice on the 48 h taste preference test and 10 mM citric acid (Sour). As stevioside tastes 300 times sweeter than sucrose and 3% sucrose is preferred by WT mice to a great extent, we used 0.01% or 124 μM stevioside, and similarly 124 μM rebaudioside A or steviol^29^,^47^. The same animals were used in the control condition and the condition with steviol added and the indicated significance is the result of a paired *t*-test from two individual experiments with 24–48 animals per group each. For the brief access drinking tests, we used a custom-build lick-o-meter^49^. The mice were deprived from water for 23 h before the experiment. Individual mice were placed in a cage equipped with a copper grounding platform under two metal drinking spouts connected to an analogue/digital converter. The drinking spouts were isolated except for the tip so only tongue licks are recorded as a positive step of 25–150 mV. Custom software recorded the junction potential at 200 Hz. We analysed potentials exceeding 25 mV as a licking event. Licking events were recorded for 60 min. We analysed the total number of licks and the fraction of licks from each bottle (per mouse) over the complete time course to measure the 1 h preference. The first 5 min were analysed for initial taste preference. For each mouse, the total number of licks was normalized to avoid unequal contribution of extreme individuals. These incremental licks were pooled and divided by the number of animals in the group to yield the data in Fig. 5b–g and Supplementary Figs 7–9. Statistical significance was assessed with paired or two-sample *t*-tests on the normalized fractions that each mouse drank at the end of 5 min (Fig. 5f) or 60 min (Supplementary Fig. 6a,b). The variance between individual mice in the same condition is displayed with the data spread and the 25–75% data-interval boxplot, indicating the mean and median of each group. The amount of licks in each condition for each mouse is represented in Supplementary Fig. 6c–e for the 60 min lick-o-meter test and in Supplementary Fig. 6f for the first 5 min.

### Data analysis and statistics

We used Matlab R2012a, OriginPro 8.6, Microsoft Excel and Igor Pro 6.34 for data analysis. Values deviating>3 × s.d. of the population mean were considered outliers. Normality was confirmed with the Shapiro–Wilk test (95% confidence level). For significance testing, we used two-tailed paired or unpaired *t*-test, unless otherwise indicated. For equivalence testing, we used the two one-sided tests approach and withheld the largest *P*-value. Data represent mean±s.e.m. Significance levels are indicated in the figures as *P*>0.05, not significant; *P<0.05; **P<0.01; ***P<0.001.

### Data availability

The data that support the findings of this study are available upon request from the corresponding author.

## Additional information

**How to cite this article:** Philippaert, K. *et al*. Steviol glycosides enhance pancreatic beta-cell function and taste sensation by potentiation of TRPM5 channel activity. *Nat. Commun.* **8,** 14733 doi: 10.1038/ncomms14733 (2017).

**Publisher's note:** Springer Nature remains neutral with regard to jurisdictional claims in published maps and institutional affiliations.

## Supplementary Material

Supplementary Information Supplementary figures and supplementary tables.
