# Supplementary material for: Steviol glycosides enhance pancreatic beta-cell function and taste sensation by potentiation of TRPM5 channel activity
Source: Nat Commun. 2017 Mar 31;8:14733. doi: 10.1038/ncomms14733 (PMC5380970; doi:10.1038/ncomms14733)
Supplement: Supplementary Information — Supplementary figures and supplementary tables. [file ncomms14733-s1.pdf]

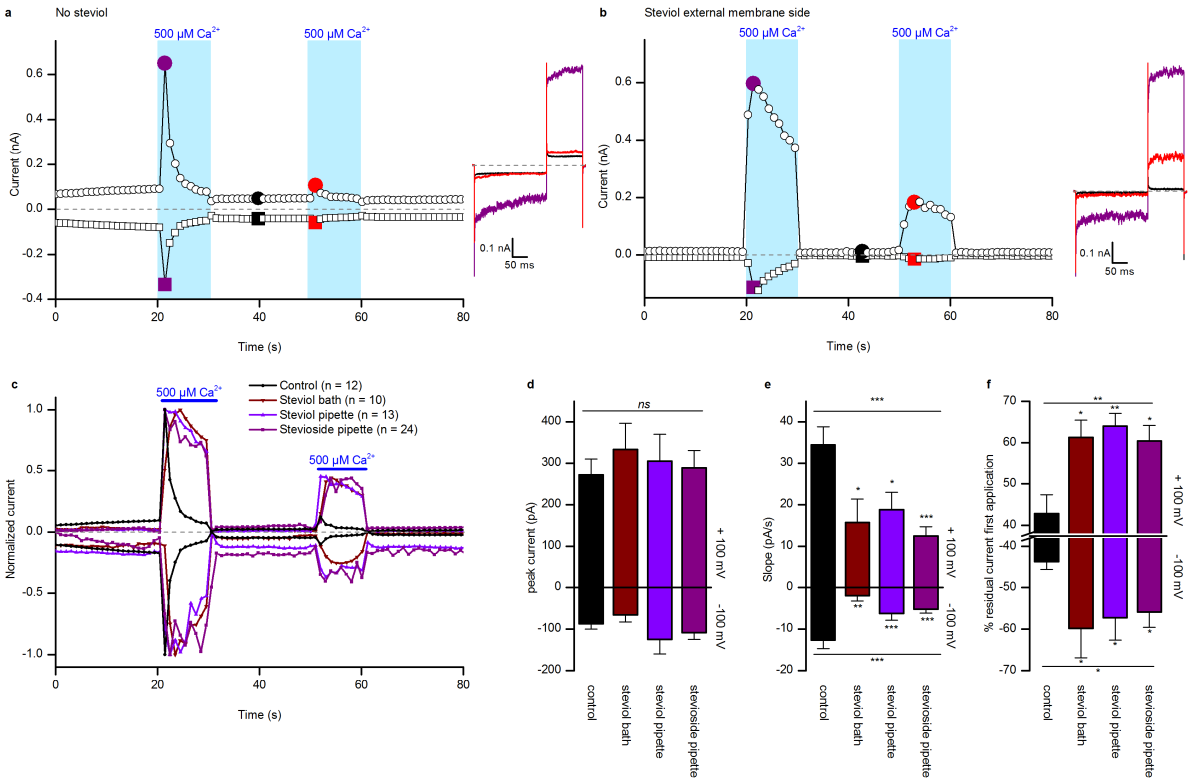

Supplementary Figure 1: **Steviol and stevioside potentiate TRPM5 in a cell-free environment.** (**a**) TRPM5 currents are activated in inside-out patches during application of 500 µM Ca^{2+} at the intracellular membrane side. Example trace of the steady-state current extracted at +100 mV and -100 mV indicating the fast rundown of the calcium-activated, TRPM5 currents. (right) Traces at the time-points indicated in the time-trace, peak calcium-induced current, current in calcium-free conditions and the peak currents during a second application of calcium. (**b**) Example as **a** but with 10 µM steviol at the external membrane side. Note the slower rundown of the calcium induced TRPM5 currents. (**c**) Traces of **a** (control), **b** (steviol external), internal steviol application or external application of stevioside, normalized to the maximal inward or outward current. (**d**) Maximum calcium-induced peak current. (**e**) Slope of the rundown of the currents is significantly smaller in the presence of steviol or stevioside (global ANOVA, Bonferroni test for means comparison). (**f**) The percentage of current left after 10 s of calcium application, compared to the maximal current. The bar graphs represent the average ± s.e.m..

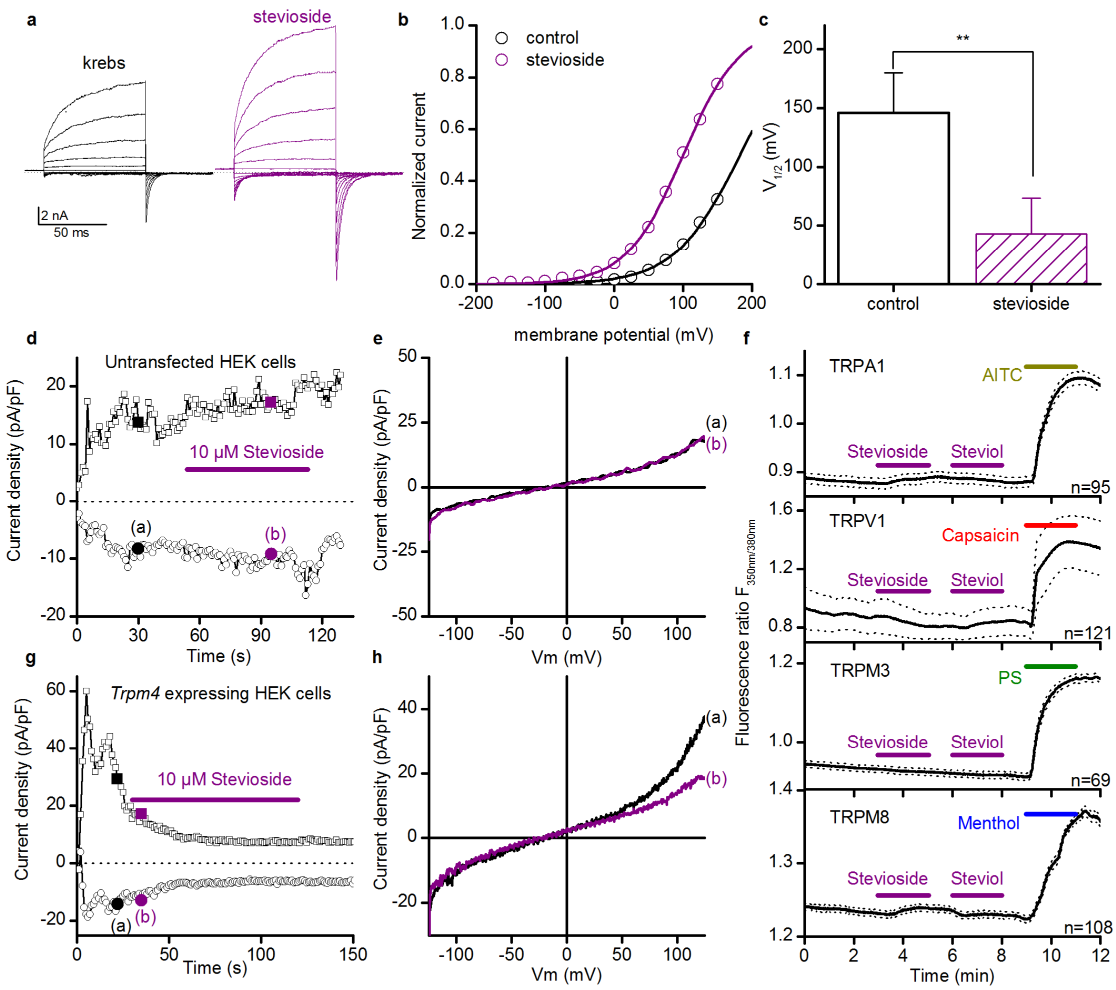

Supplementary Figure 2: **Mechanism of TRPM5 activation by stevioside and its selectivity.** (**a**) Representative current traces during a voltage step protocol in control conditions (left, black) and during stevioside application (right, purple). The dotted line represents the 0 current level. (**b**) Representative examples of the normalized IV relation (from **a**) in control conditions and during application of 10 µM stevioside. (**c**) Average ± s.e.m. (n = 8 cells, paired t-test, P = 0.009) voltage for half-maximal activation in control conditions and in the presence of 10 µM stevioside. (**d**) Typical time trace of the stevioside evoked current in non-transfected HEK cells. (**e**) Typical I-V curves before and during stevioside application of the time trace in (**d**). (**f**) Calcium imaging experiments on HEK cells expressing *Trpa1, Trpv1, Trpm3* or *Trpm8*, as indicated (average ± s.e.m.). Note the lack of effect of 10 µM stevioside or steviol, and, instead, robust Ca^{2+} signals when designated agonists for the respective channels are applied. (**g**) Example of a patch recording of *Trpm4* expressing HEK cells. Note the lack of effect of 10 µM stevioside. (**h**) Representative I-V curves of the experiment in (**g**).

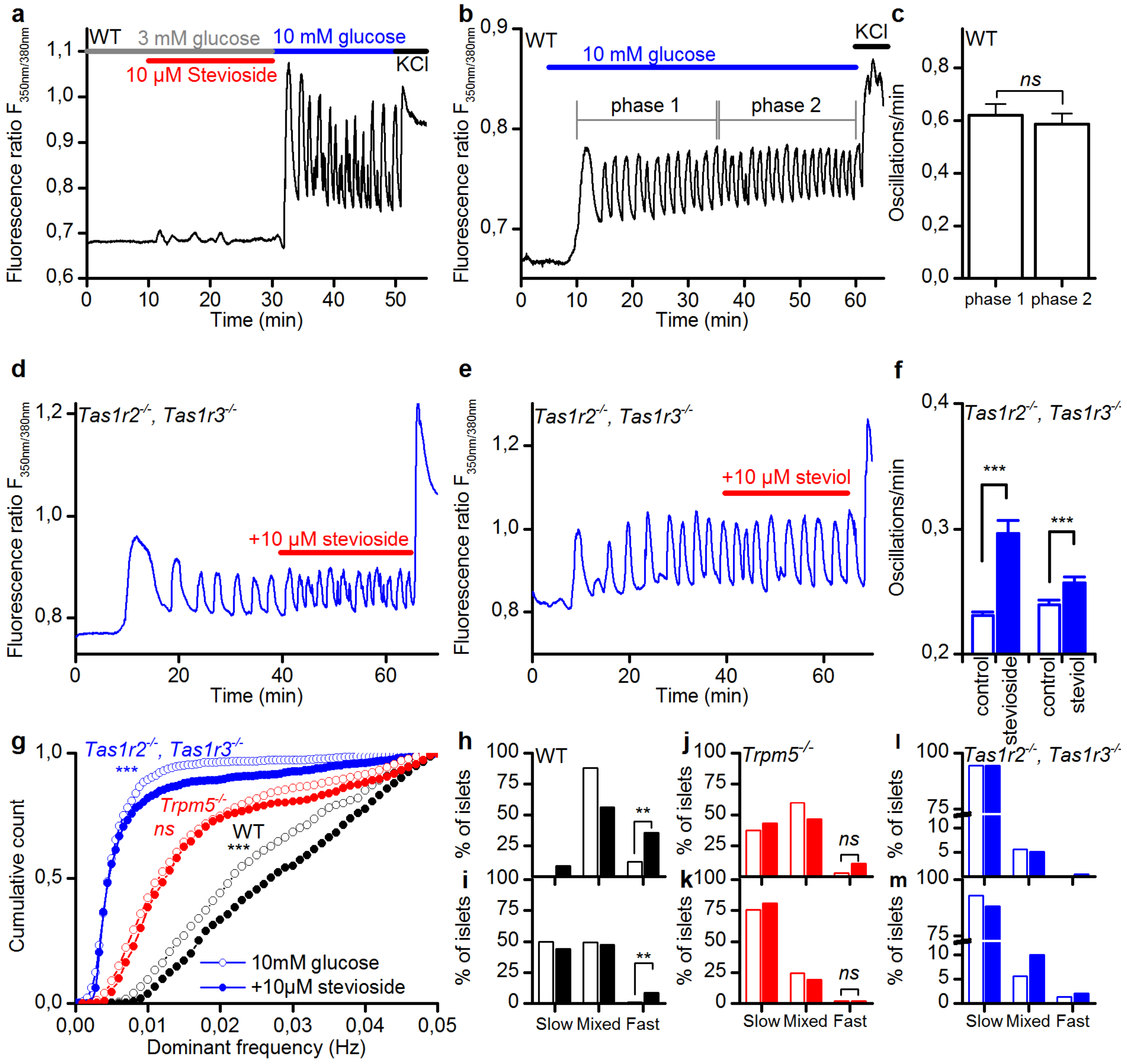

Supplementary Figure 3: **Calcium imaging on isolated pancreatic islets.** (**a**) Stevioside alone is not sufficient to initialize calcium oscillations in low (3 mM) glucose conditions. (**b**) In WT islets, the glucose induced calcium oscillations occur at a constant frequency without application of stevioside. (**c**) Average ± s.e.m. calcium oscillation frequency during the first phase of the experiment and the second phase of the experiment is not different (paired sample t-test), both averages are equivalent within 0.05 osc./min (two one-sided tests for equivalence). (**d**) Representative glucose-induced calcium oscillations in islets from *Tas1r2*$^{-/-}$, *Tas1r3*$^{-/-}$ mice. 10 µM stevioside was applied as indicated. (**e**) As **d** with application of steviol instead of stevioside. (**f**) The average ± s.e.m. oscillation frequency of *Tas1r2,Tas1r3*$^{(-/-)2}$ islets as in **d** and **e** (Stevioside: n = 395 islets from 10 mice, P = 4.95x10^{-10} paired t-test. Steviol: n = 280 islets from 5 mice, P = 2.8x10^{-8} paired t-test)

before or during stevioside or steviol perfusion in high glucose conditions. (**g**) The cumulative distribution of dominant calcium oscillation frequencies in WT, *Trpm5*$^{-/-}$ and *Tas1r2,Tas1r3*$^{(-/-)2}$ islets in the presence of stevioside compared to control conditions. (**h**) Division of the WT islets in slow (dominant frequency <0.015 Hz), fast (dominant frequency >0.015 Hz) and mixed (dominant oscillation frequencies in both parts of the spectrum) oscillating islets in the presence (filled bars) or absence (open bars) of stevioside or (**i**) steviol. (**j**) Division of the *Trpm5*$^{-/-}$ islets in slow, fast and mixed oscillating islets in the presence or absence of stevioside or (**k**) steviol. (**l**) Division of the *Tas1r2*$^{-/-}$*, Tas1r3*$^{-/-}$ islets in slow, fast and mixed oscillating islets in the presence or absence of stevioside or (**m**) steviol.

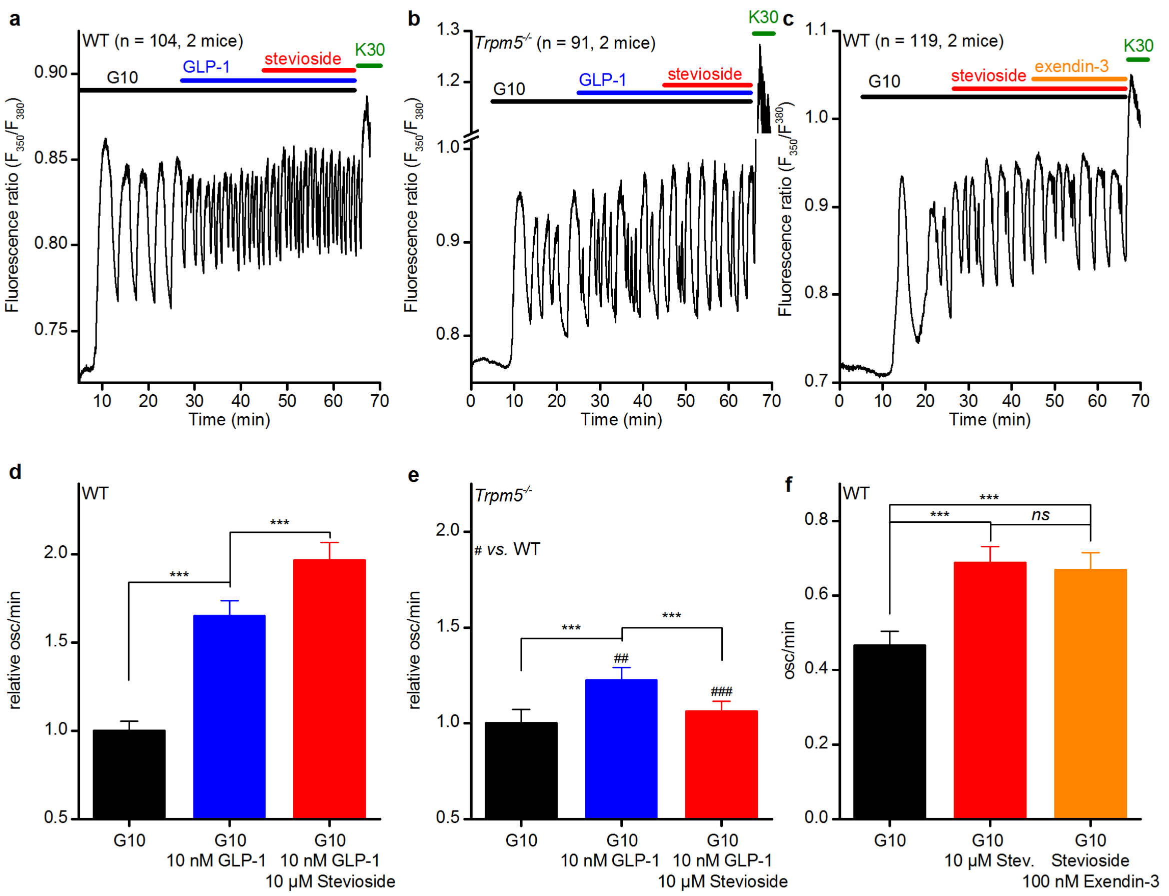

Supplementary Figure 4: **Physiological modulation of calcium oscillations in islets.** (**a**) Example trace of the intracellular calcium in a WT islet exposed to G10, 10 nM GLP-1 and 10 µM stevioside illustrating that GLP-1 and stevioside have complementary effects. (**b**) Example trace of a *Trpm5*$^{-/-}$ islet exposed to G10, 10 nM GLP-1 and 10 µM stevioside, showing reduced effects of GLP-1 and no effect of stevioside. (**c**) Example trace of a WT islet exposed to G10, 10 µM stevioside and 100 nM exendin-3 a competitive GLP-1R antagonist. Inhibiting GLP1-R signalling does not affect the effect of stevioside on calcium oscillations. (**d**) Average ± s.e.m. of the effect of GLP-1 and stevioside on WT islets (paired sample t-test). (**e**) Average ±s.e.m. of the effect of GLP-1 and stevioside on *Trpm5*$^{-/-}$ islets (paired sample t-test). # indicates the differences between WT and *Trpm5*$^{-/-}$ islets (two sample t-test). (**f**) Average ± s.e.m. showing the lack of effect of exendin-3 on the stevioside-induced potentiation of calcium oscillations in WT islets (paired sample t-test).

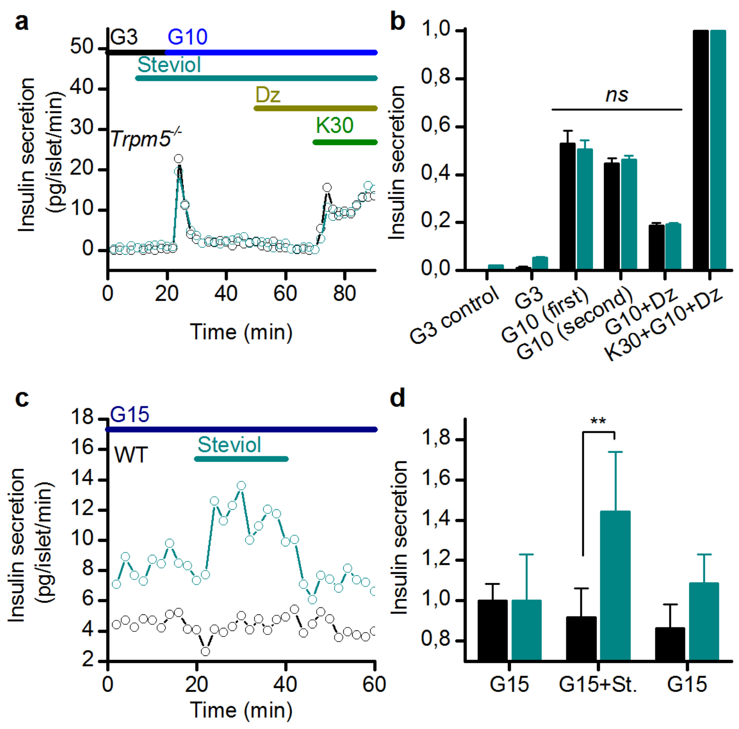

Supplementary Figure 5: **Stevioside stimulates glucose induced insulin secretion without affecting insulin tolerance.** (**a**) Example trace of insulin secretion during perfusion experiments of *Trpm5*$^{-/-}$ islets without steviol (black) or with steviol supplementation at 10 min in 3 mM glucose (G3), 10 mM glucose (G10) and with 100 µM Diazoxide (Dz) and 30 mM KCl (K30). (**b**) Total insulin secretion in different conditions relative to the K30 stimulus. First GIIS is the initial insulin peak between 20’ and 30’ in (**a**) and the second phase between 30’ and 50’. No difference between control and steviol conditions with a two-way ANOVA (average ± s.e.m., n = 178 islets, from 5 mice in 4 perfusion experiments). (**c**) Steviol (10 µM) potentiates the glucose induced insulin secretion (15 mM glucose – G15) in WT islets. There is no potentiation of GIIS in the absence of steviol (black). (**d**) Average potentiation of G15-induced insulin secretion with steviol application (n = 5 experiments, 230 islets) and with vehicle application (n = 3 experiments, 120 islets from 22 mice in total, average ± s.e.m., t-test).

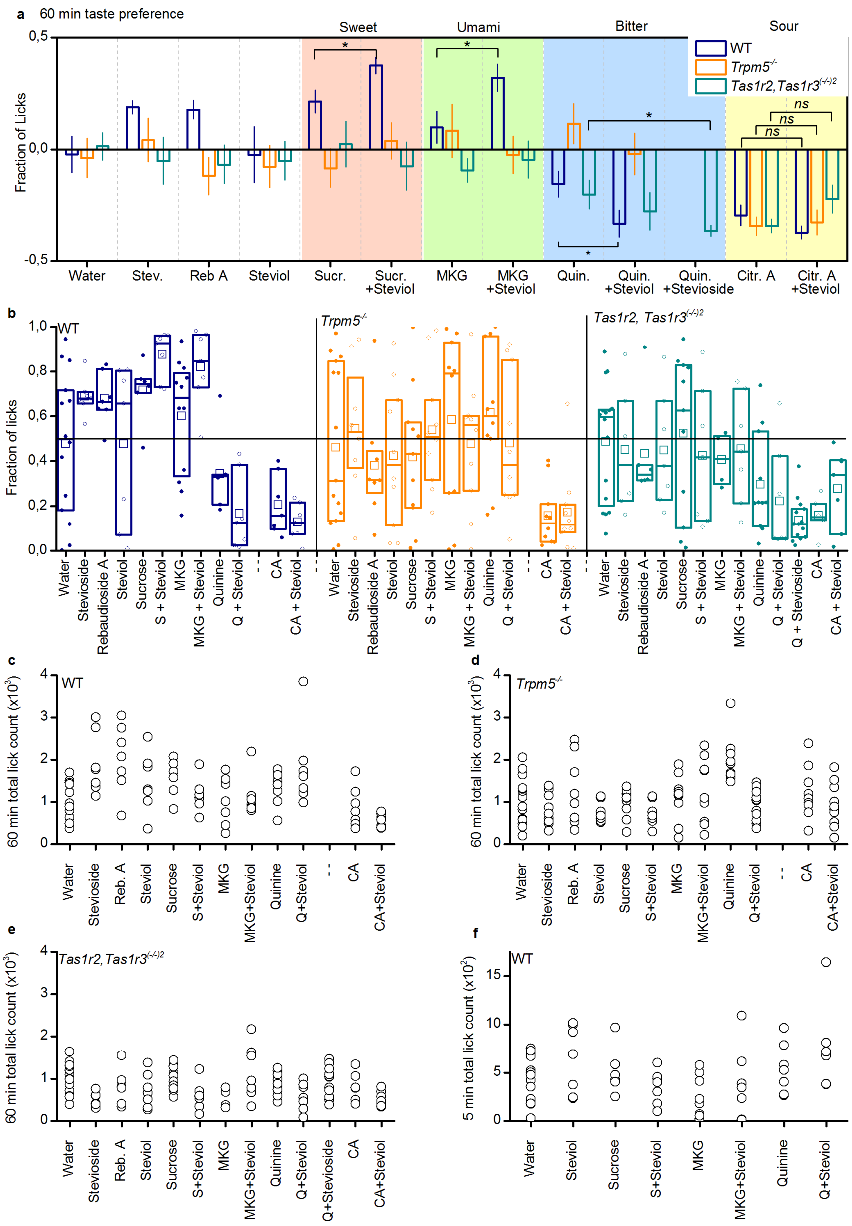

Supplementary Figure 6: **Lick-o-meter preferences and lick count.** (**a**) 1 hour licking preference for a bottle containing the indicated tastant compared to water for WT, *Trpm5*$^{-/-}$ and *Tas1r2,Tas1r3*$^{(-/-)2}$ mice, represented as average ± s.e.m. relative fraction of licks during 60 minutes (t-test). (**b**) Variation in preference from (**a**) the circles indicate the preference of each individual mouse with the 25%-75% interval in the boxplot with the median (line) and mean (squares) indicated. Conditions with high preference typically display the lowest variance. (**c**) Sum of the licks from both bottles during the two bottle lick-o-meter test for 60 min from WT, (**d**) *Trpm5*$^{-/-}$ and (**e**) *Tas1r2,Tas1r3*$^{(-/-)2}$ mice. Each circle represents one mouse. (**f**) Lick count from the first 5 min of the lickometer test from the conditions in **Figure 5 f**. See also **Supplementary Figure 7, 8** and **9** for the full time course.

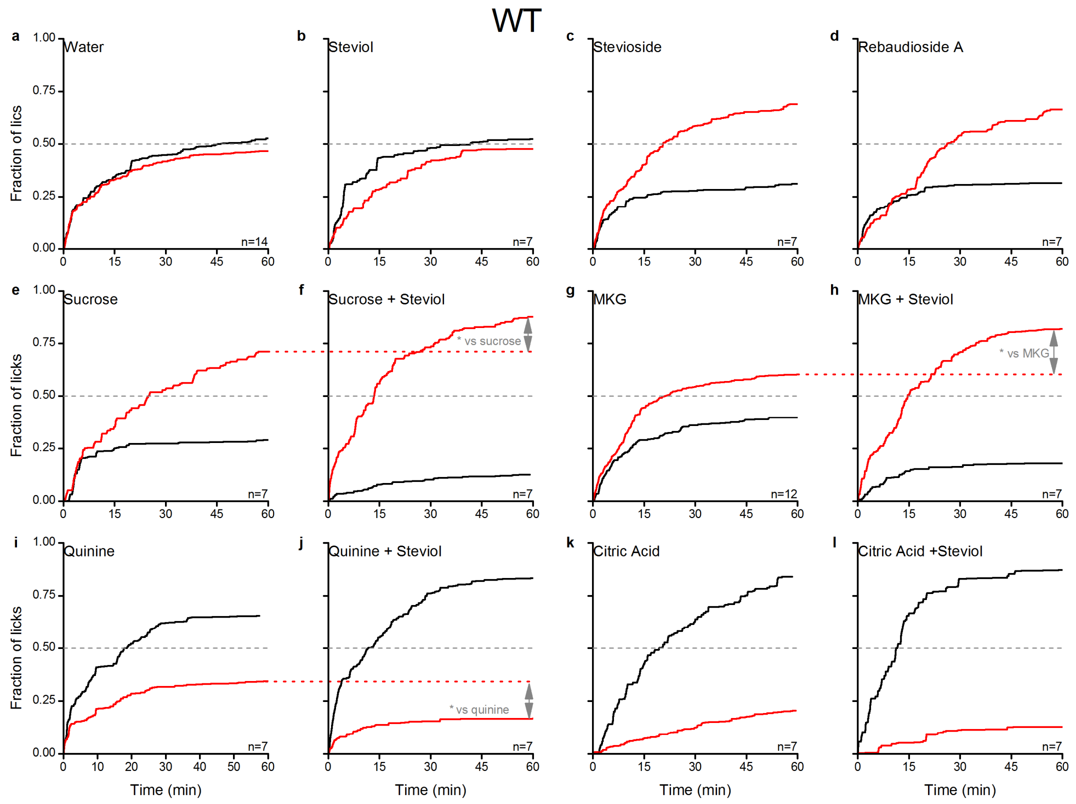

Supplementary Figure 7: **Short term taste preference in WT mice.** (Supplementary Figure to **Figure 5b-g**) Normalized fraction of licks from the bottle containing water (black) or the indicated tastant (red) over 60 min after 23 h water deprivation for WT mice. (**a**) Indifference for water *vs.* water. (**b**) Indifference for 124 µM steviol *vs.* water. (**c**) Preference for 124 µM stevioside *vs.* water. (**d**) Preference for 124 µM rebaudioside A *vs.* water. (**e**) Preference for 1% sucrose *vs.* water and (**f**) increased preference for 1% sucrose with 124 µM steviol *vs.* water (paired sample t-test). (**g**) Preference for 150 mM MKG *vs.* water and (**h**) increased preference for 150 mM MKG with 124 µM steviol *vs.* water (two sample t-test). (**i**) Avoidance for 100 µM quinine *vs.* water and (**j**) increased avoidance for 100 µM quinine with 124 µM steviol *vs.* water (two sample t-test). (**k**) Avoidance for 10 mM citric acid vs. water and (**l**) similar avoidance for 10 mM citric acid with 124 µM steviol *vs.* water.

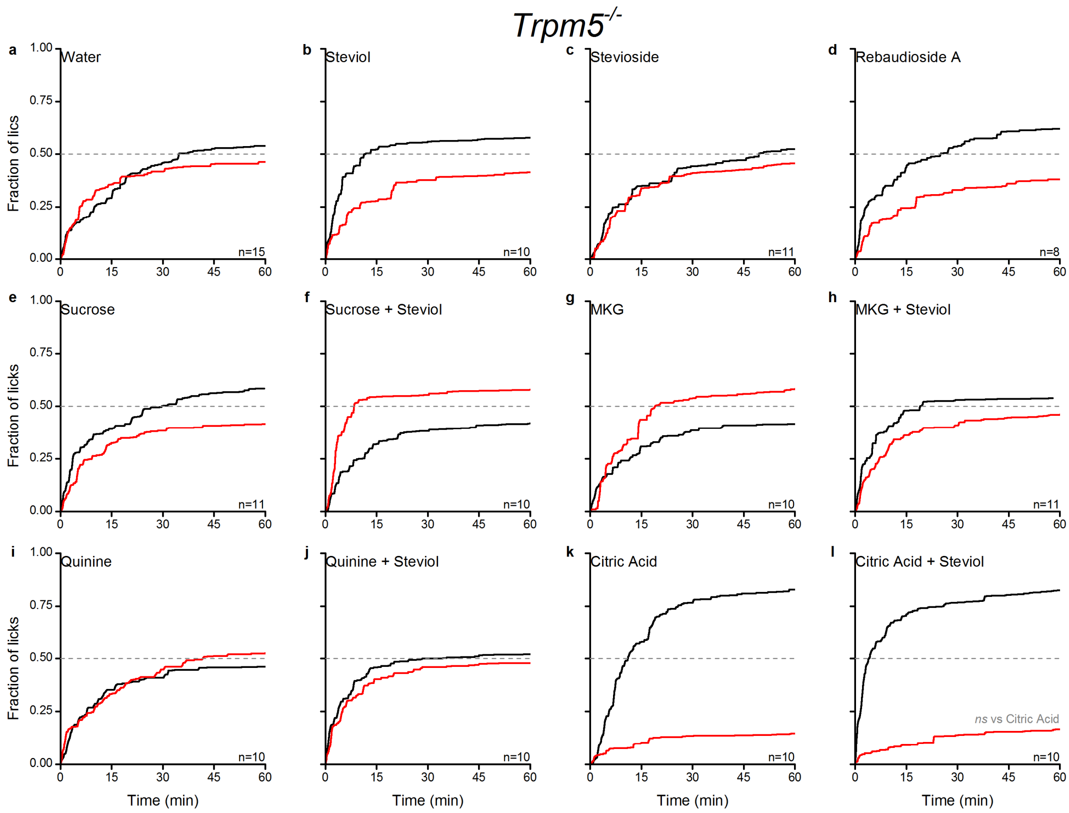

Supplementary Figure 8: **Short term taste preference in *Trpm5*$^{-/-}$ mice.** (Supplementary Figure to **Supplementary Figure 6**) Normalized fraction of licks from the bottle containing water (black) or the indicated tastant (red) over 60 min after 23 h water deprivation for *Trpm5*$^{-/-}$ mice. (**a**) Indifference for water *vs.* water. (**b**) Indifference for 124 µM steviol *vs.* water. (**c**) Indifference for 124 µM stevioside *vs.* water. (**d**) Indifference for 124 µM rebaudioside A *vs.* water. (**e**) Indifference for 1% sucrose *vs.* water and (**f**) indifference for 1% sucrose with 124 µM steviol *vs.* water. (**g**) Indifference for 150 mM MKG *vs.* water and (**h**) indifference preference for 150 mM MKG with 124 µM steviol *vs.* water. (**i**) Indifference for 100 µM quinine *vs.* water and (**j**) indifference for 100 µM quinine with 124 µM steviol *vs.* water. (**k**) Avoidance for 10 mM citric acid *vs.* water and (**l**) similar avoidance for 10 mM citric acid with 124 µM steviol *vs.* water (paired sample t-test).

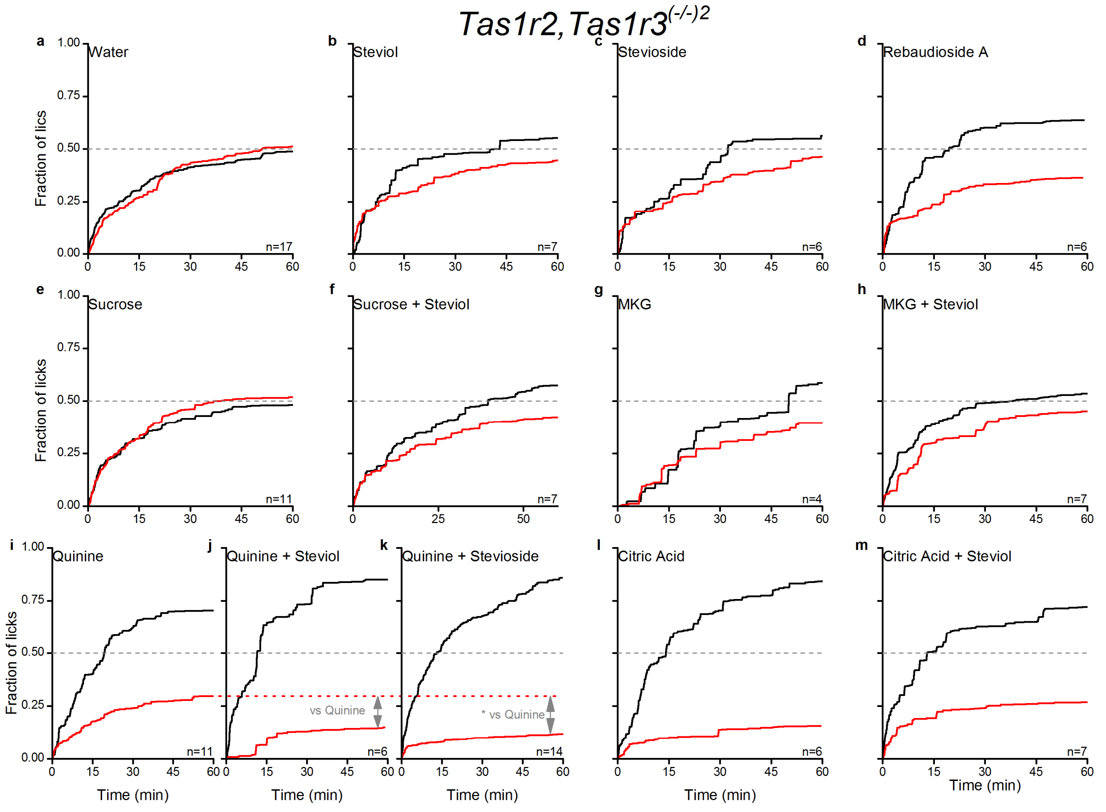

Supplementary Figure 9: **Short term taste preference in *Tas1r2, Tas1r3*$^{(-/-)2}$ mice.** (Supplementary Figure to **Supplementary Figure 6**) Normalized fraction of licks from the bottle containing water (black) or the indicated tastant (red) over 60 min after 23 h water deprivation for *Tas1r2, Tas1r3*$^{(-/-)2}$ mice. (**a**) Indifference for water *vs.* water. (**b**) Indifference for 124 µM steviol *vs.* water. (**c**) Indifference for 124 µM stevioside *vs.* water. (**d**) Indifference for 124 µM rebaudioside A *vs.* water. (**e**) Indifference for 1% sucrose *vs.* water and (**f**) indifference for 1% sucrose with 124 µM steviol *vs.* water. (**g**) Indifference for 150 mM MKG *vs.* water and (**h**) indifference for 150 mM MKG with 124 µM steviol *vs.* water. (**i**) Avoidance for 100 µM quinine *vs.* water and (**j**) increased avoidance for 100 µM quinine with 124 µM steviol *vs.* water or (**k**) 100 µM quinine with 124 µM stevioside (two sample t-test). (**l**) Avoidance for 10 mM citric acid *vs.* water and (**m**) similar avoidance for 10 mM citric acid with 124 µM steviol *vs.* water.

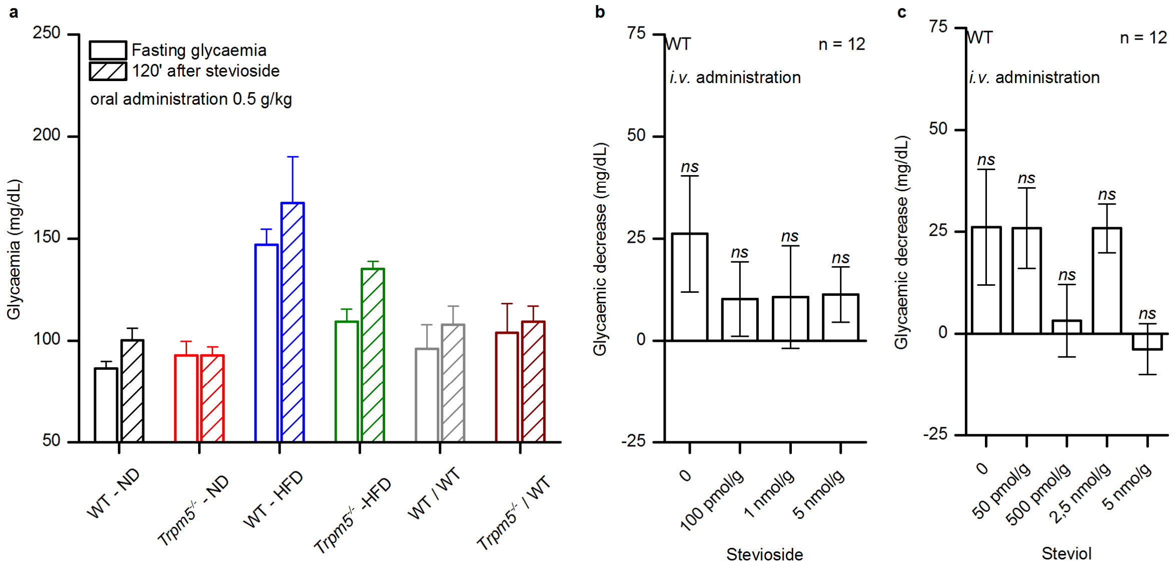

Supplementary Figure 10: **Administration of stevioside or steviol does not induce hypoglycaemia.** (**a**) Glycaemia after overnight fasting glycaemia (open bars) and glycaemia 2 hours after the oral administration of 0.5 g/kg stevioside (hashed bars). These values (average ± s.e.m.) indicate no induction of hypoglycaemia in before the GTT experiments in **Figure 6a-f**. (**b**) The difference in glycaemia after 4 hours of fasting and 1 hour after *i.v.* injection of stevioside or vehicle via the tail vein in WT animals. (**c**) as **b** but different doses of steviol were injected. One sided t-test *vs.* 0, n = 10 mice/condition.

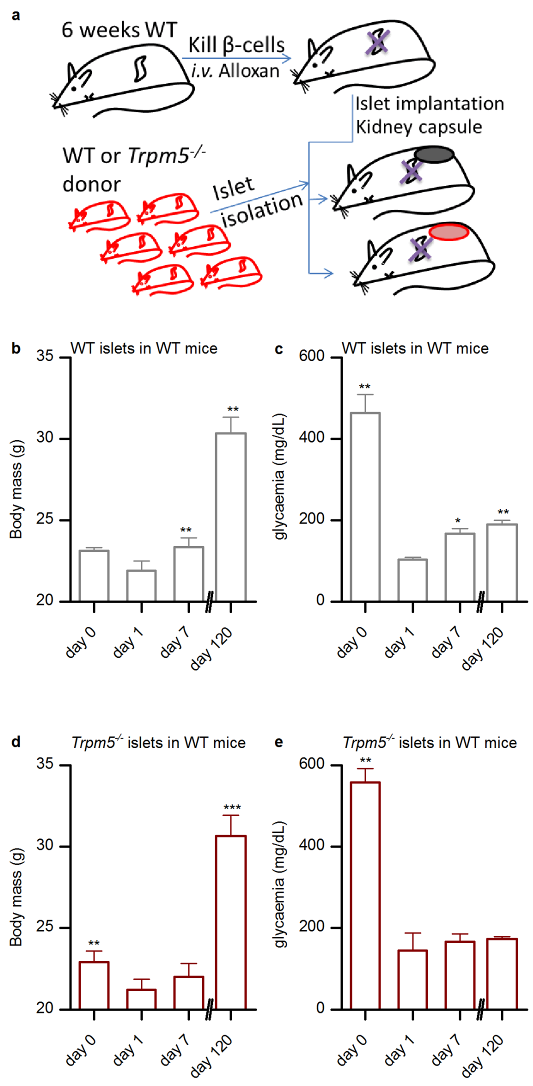

Supplementary Figure 11: **Body mass and glycaemia before and after islet transplantations.** (**a**) Schematic protocol of transplantation experiments creating WT animals with functional *Trpm5*$^{-/-}$ islets. (**b**) Body mass (average ± s.e.m.) of WT animals that received WT islets at day 0, before surgery and at various time points after surgery. After initial body mass loss the mice quickly recovered to the same weight as before surgery. Significances indicated are the result of a pairwise t-test *vs.* the value at day 1. (**c**) Hyperglycaemia before the transplantation indicates alloxan-mediated destruction of the native β-cells in the acceptor mouse. Recovery to normoglycaemic values is seen as soon as 1 day after the surgery, and is maintained for several months (average ± s.e.m.). (**d**), (**e**) As **b** and **c** but for the mice receiving *Trpm5*$^{-/-}$ islets.

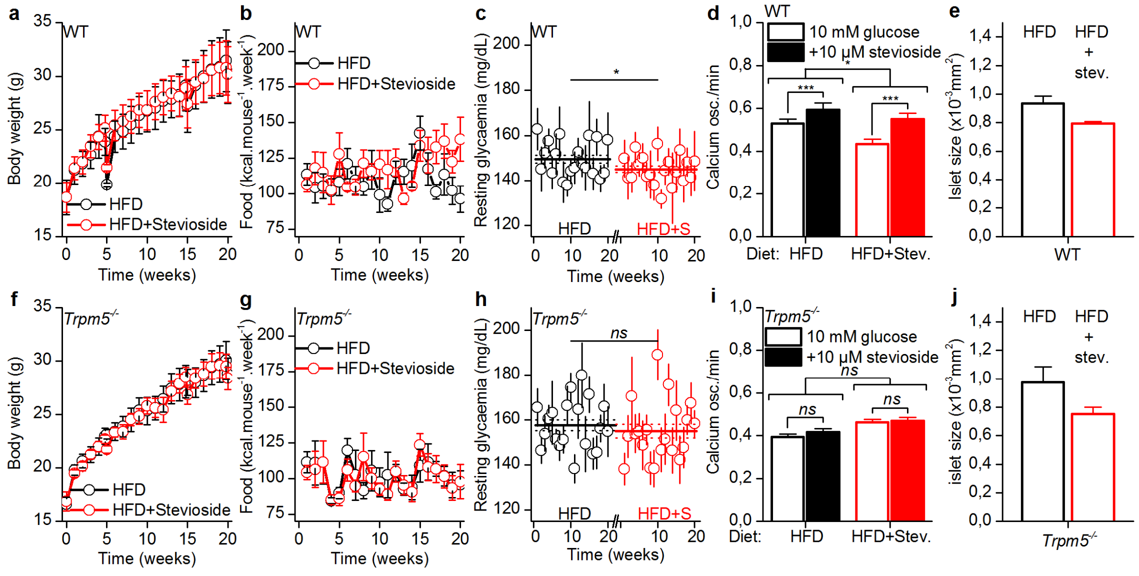

Supplementary Figure 12: **Physiological parameters of mice during and after 20 weeks of HFD**. (**a**) Body weight evolution (average ± s.e.m.) of WT mice on a HFD (black) or HFD supplemented with daily stevioside (25 $mg.kg^{-1}.day^{-1}$) (red) over the course of 20 weeks. (**b**) Weekly food intake of WT mice during 20 weeks of HFD with or without stevioside in the diet, as indicated (n = 8 mice/group average ± s.e.m.). (**c**) Resting blood glucose values of the same WT mice on HFD with or without stevioside treatment (weekly average ± s.d., circles and error bars, global average horizontal line ± s.e.m. dashed lines). (**d**) Frequency of calcium oscillations of pancreatic islets isolated from WT mice after 20 weeks on a HFD with (red) or without (black) stevioside treatment. The oscillations were recorded during perfusion with 10 mM glucose (open bars) or 10 mM glucose + 10 µM stevioside (filled bars n = 90-95 islets from 4 mice, average ± s.e.m., paired t-test within groups, two sample t-test on the difference between groups). (**e**) Islet size (average ± s.e.m.) of the measured islets from WT mice on HFD with or without stevioside. (**f**) Body weight of *Trpm5*$^{-/-}$ mice (**g**) Weekly food intake of *Trpm5*$^{-/-}$ mice (n = 8 mice/group average ± s.e.m.). (**h**) Average resting glucose of *Trpm5*$^{-/-}$ mice. (**i**) Calcium oscillation frequencies as in (**d**) but from *Trpm5*$^{-/-}$ mice (n = 70-80 islets from 4 mice, average ± s.e.m.). (**j**) Islet size of *Trpm5*$^{-/-}$ islet on HFD with or without stevioside.

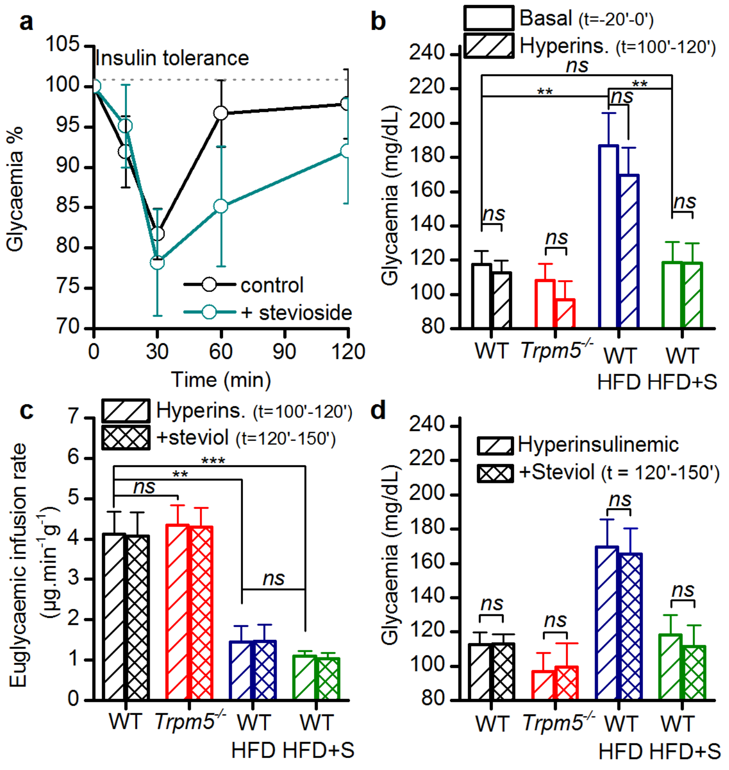

Supplementary Figure 13: **SGs do not influence the insulin tolerance in WT and *Trpm5*$^{-/-}$ mice.** (**a**) Percentage change in the glycaemia during an insulin tolerance test of 10 WT animals in control conditions and 10 WT animals after 3 weeks of exposure to 124 µM stevioside in their drinking water. No difference in insulin tolerance was observed (average ± s.e.m., two sample t-test). (**b**) Basal and euglyemic glycaemia (average ± s.e.m.) from the experiment in **Figure 5 g-j**. Mice on HFD are hyperglycaemic compared to mice on a normal diet or mice with HFD + steviol (t-test). (**c**) Euglycaemic infusion rate (average ± s.e.m.) indicating insulin sensitivity. Mice on a HFD or HFD+S are insulin resistant compared to normal WT animals. Stevioside supplementation to the diet does not change the insulin sensitivity in animals on a HFD (t-test). (**d**) Euglycaemic glycaemia (average ± s.e.m.) and the lack of effect caused by acute steviol infusion during hyperinsulinemic euglycaemic clamp (paired t-test).

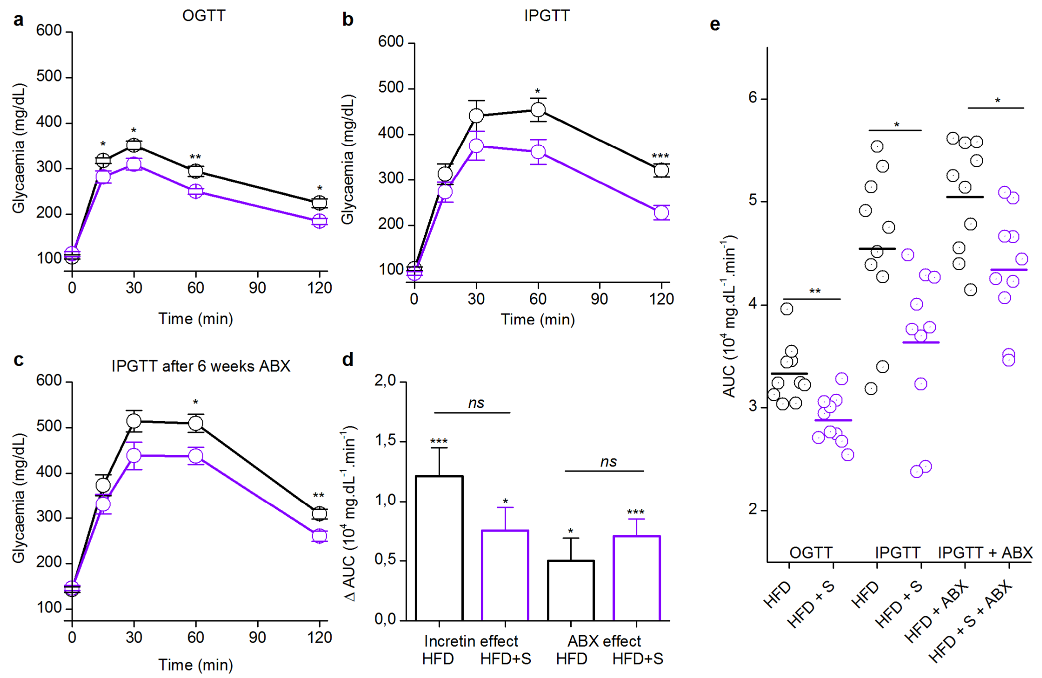

Supplementary Figure 14: **Stevioside acts independent from the incretin pathway or the gut microbiome.** (**a**) Oral GTT (OGTT) of 10 male WT mice after 20 weeks on HFD (black) and 10 age-matched male WT mice that received stevioside (purple). Indicated significances are the result of a two-sample t-test between the groups at the same time-point. Individual points are represented as average ± s.e.m.. (**b**) Intra-Peritoneal (IP) GTT of the same mice, one week later. (**c**) IPGTT of the same mice in (**a**) and (**b**) after 6 weeks of antibiotics (ABX). (**d**) The difference in AUC attributed to the incretin effect (difference between (**a**) and (**b**)) and the effect of the antibiotics treatment (difference between (**b**) and (**c**)). Average ± s.e.m., one-sample t-test *vs.* 0 indicated for each group. Non-significant differences between groups were obtained with a two-sample t-test. (**e**) AUC of the GTT in (**a**, **b** and **c**) and comparison (two-sample t-test) of stevioside treated (purple) and control mice (black).

Supplementary Table 1: **Supplementary information to Figure 4**

	**WT** average±sem Control	**10 µM Steviol**	***Trpm5*$^{-/-}$** average±sem Control	**10 µM Steviol**
5 mM Glucose	0.20±0.05	0.32±0.11	0.33±0.11	0.31±0.06
20 mM Glucose	1.52±0.27	1.38±0.10	1.54±0.28	1.65±0.15
10 mM Glucose + 30 mM KCl	1.55±0.26	1.46±0.15	1.50±0.13	1.56±0.11

Static insulin secretion. Insulin release as % of content in islets exposed to low glucose (5 mM), high glucose (20 mM) and 30 mM KCl with or without 10 µM steviol. There are no significant differences in insulin release upon addition of steviol during these supraphysiological stimulations (two sample t-test).

Supplementary Table 2**: Supplementary information to Figure 7**

	WT control	WT stevioside	*P =*	*Trpm5*$^{-/-}$ control	*Trpm5*$^{-/-}$ stevioside	*P =*
**age (weeks)**	7	7	1	7	7	1
**Start weight (g)**	18.7±1.73	18.7±1.49	0.99	16.6±0.68	16.8±0.95	0.84
**Tibial length (mm)**	1.98±0.040	1.98±0.038	1	1.93±0.080	1.87±0.026	0.15

Size, weight and age of WT and *Trpm5*$^{-/-}$ mice before 20 weeks HFD (from **Fig. 7**). No significant differences in age, body weight or tibial length of the mice within either genotype (n = 8 mice/group). The represented values are the average ± s.e.m. P value represents the result of a two-tailed two sample t-test between control and stevioside treated group.

Supplementary Table 3: **Supplementary information to Figure 8.**

**Body weight (g)**<br>**Age (weeks)**	7	17	22	P-value week 17 vs 22
**Stevioside-continuous (Group 1; n = 8 mice)**	24.80 ± 0.91	32.48 ± 1.39	33.80 ± 2.36	**P = 0.0135**
**Stevioside-stop (Group 2; n = 10 mice)**	20.24 ± 0.32	34.42 ± 1.38	43.77 ± 2.05	**P = 4.4 x 10^{-6}**
**Control (Group 3; n = 8 mice)**	23.21 ± 0.34	42.60 ± 1.19	47.38 ± 1.79	**P = 0.0014**

**Fasting glycaemia (mg/dL)**<br>**Age (weeks)**	7	17	22	P-value week 17 vs 22
**Stevioside-continuous (Group 1; n = 8 mice)**	84.6±6.6	133.9±6.4	112.6±14.3	P = 0.24
**Stevioside-stop (Group 2; n = 10 mice)**	90.8±2.8	99.8±4.2	117.6±6.7	**P = 0.028**
**Control (Group 3; n = 8 mice)**	81.9±3.9	129.0±16.0	142.0±13.2	P = 0.85

**Glycaemia at 120' (mg/dL)**<br>**Age (weeks)**	7	17	22	P-value week 17 vs 22
**Stevioside-continuous (Group 1; n = 8 mice)**	124.8±7.6	191.1±10.4	186.3±28.8	P = 0.83
**Stevioside-stop (Group 2; n = 10 mice)**	134.1±6.5	196.3±13.9	308.8±26.2	**P = 0.001**
**Control (Group 3; n = 8 mice)**	119.2±9.5	270.4±25.8	299.7±43.0	P = 0.39

Physiological parameters of mice on a HFD with or without stevioside. The body weight, fasting glycaemia and glycaemia 120' after glucose challenge of mice on a HFD diet (from **Fig. 8**). The first group is treated with stevioside (Group 1), in the second group stevioside treatment was ceased at the age of 17 weeks (Group 2) and the third group did not receive stevioside (Control - Group 3; as indicated in **Fig. 8a**). The P values indicate the results of a paired t-test between data obtained at the age of 17 weeks *vs.* 22 weeks.
